# CDELDA: A Content-Based Dual-Encoder for Cold-Start lncRNA–Disease Association Prediction

**DOI:** 10.3390/biomedicines14092121

**Published:** 2026-09-19

**Authors:** Sanguk Kim, Taehyeon Yun, Jihwan Ha

**Affiliations:** 1 Department of Data Engineering, Pukyong National University, Busan 48513, Republic of Korea; rlatkddnr122@pukyong.ac.kr (S.K.); ytae1014@pukyong.ac.kr (T.Y.); 2 Major of Big Data Convergence, Division of Data Information Science, Pukyong National University, Busan 48513, Republic of Korea

**Keywords:** long non-coding RNA, lncRNA–disease association, cold-start prediction, inductive evaluation, dual-encoder, representation learning, deep learning, benchmarking

## Abstract

**Background/Objectives:** An important challenge in long non-coding RNA (lncRNA)–disease association prediction is prioritizing candidates when neither target node has association records in the training data, even though content features are available. This study aimed to develop and evaluate a content-based model for this both-cold setting. **Methods:** We developed CDELDA, a dual-encoder model combining lncRNA sequence, structure, and expression features with disease text, semantic similarity, and ontology-structure features. CDELDA was compared with five baseline methods on a normalized benchmark comprising 1662 lncRNAs, 220 diseases, and 9920 positive associations. Evaluation covered warm, lncRNA-cold, disease-cold, and both-cold settings, with association-derived inputs constructed from training labels. Disease-macro area under the receiver operating characteristic curve (AUROC) was the main comparison metric, complemented by average precision and top-ranked retrieval metrics. Feature ablation, disease-feature perturbation, sensitivity analyses, and literature-based case studies further characterized model performance. **Results:** Under both-cold evaluation, CDELDA achieved a mean disease-macro AUROC of 0.6617, compared with 0.6123 for SIMCLDA, the highest-scoring literature baseline on this metric. Relative performance varied across evaluation settings and metrics. Removing expression features produced the largest decrease in disease-macro AUROC among individual lncRNA feature removals. Reassigning disease features reduced performance, although ranking performance was partly retained. Literature review identified functional or observational support for 14 of 20 reviewed candidate pairs. **Conclusions:** These findings suggest that CDELDA can support content-based candidate prioritization under both-cold evaluation. The predictions provide candidates for biological follow-up, with disease-specific relevance requiring independent validation.

## 1. Introduction

Long non-coding RNAs (lncRNAs) are generally defined as RNA transcripts of at least 200 nucleotides that lack protein-coding potential. Studies have identified roles for specific lncRNAs in cellular growth, gene regulation, and cancer progression, thereby motivating investigation of their disease associations [1,2]. Establishing the biological relevance of a candidate association requires experimental investigation in an appropriate cellular or disease context. Computational prediction can support this process by prioritizing candidate associations for follow-up. Resources such as RNADisease v4.0 collect reported RNA–disease associations and provide a foundation for developing and evaluating predictive methods [3].

Computational approaches to lncRNA–disease association prediction combine known associations with similarity measures and external biological features. DSCMF applies collaborative matrix factorization, whereas SIMCLDA uses inductive matrix completion to incorporate feature information into association prediction [4,5]. IPCARF combines similarity-based features with dimensionality reduction and random forest classification [6]. Graph-based approaches include VGAELDA, which combines feature representation learning with association-label propagation, and LDA-VGHB, which integrates matrix decomposition, graph representation learning, and boosting [7,8]. Related work on miRNA–disease association prediction has explored matrix factorization and completion [9,10,11], metric learning [12], and graph-based representation learning with neural collaborative filtering [13,14], offering methodological perspectives relevant to lncRNA–disease association prediction. Zhou et al. combined gradient boosting decision trees with logistic regression for miRNA–disease association prediction [15]. Across these approaches, predictive information can be derived from observed association patterns and features obtained from external resources. The availability of these information sources for the target nodes is, therefore, an important consideration in model evaluation. Related strategies have also been investigated in other biomedical tasks, including multiplex-network ranking for heterogeneous complex disease analysis [16] and indicator-regularized non-negative matrix factorization for virus–drug association prediction [17]. Multiview and multifeature fusion has also been explored for protein secondary-structure prediction [18].

An important challenge in practical association prediction arises when content features are available for an lncRNA and a disease, but neither has association records in the training data. A model must then rank their potential association without relying on association patterns directly observed for either node. Warm evaluation, which holds out pairs while retaining training associations for both endpoints, does not directly assess this task. The distinction between predicting associations among observed nodes and generalizing to nodes without training associations has also been emphasized in related biological interaction-prediction tasks [19]. Existing lncRNA–disease association methods have investigated inductive prediction and node-holdout settings [5,8]. Both-cold evaluation specifically assesses prediction when association labels involving either test node are excluded from training.

Comparing methods across these settings requires a clear distinction between information available during training and associations reserved for testing. Constructing association-derived similarities and graph features from training labels prevents held-out associations from being included in these inputs. Consistent node sets, partitions, and evaluation controls are also needed to make the comparisons interpretable. Within this framework, the present study addresses two questions: how effectively content-based representations support both-cold prediction, and which content features contribute to that performance. We examine within-disease discrimination and candidate retrieval, together with feature ablation and perturbation analyses that assess the dependence of predictions on individual inputs.

To address these questions, we present CDELDA, a content-based dual-encoder model for lncRNA–disease association prediction. The lncRNA representation combines sequence, structure, and expression features, while the disease representation combines text, semantic similarity, and ontology structure features. Separate projection networks map these representations into a shared latent space, and their inner product provides an association score. CDELDA computes node representations from available content features rather than assigning each node a free embedding learned from its association labels. The model can therefore score pairs involving nodes without training associations, provided that the required content features can be constructed.

We evaluate CDELDA alongside five baseline methods on a normalized benchmark derived from RNADisease v4.0. Gene and disease identifiers are normalized, and a common node set is retained according to the input requirements of the compared methods. Evaluation covers warm, lncRNA-cold, disease-cold, and both-cold settings, with association-derived inputs restricted to training labels. This study makes three contributions. First, it presents a multiview content-based model applicable to nodes without training associations. Second, it compares the model with five baseline methods under a common evaluation framework, with particular attention to both-cold prediction. Third, it characterizes the contribution of content features and the dependence of performance on evaluation conditions through feature ablation, disease-feature perturbation, and sensitivity analyses. Literature-based case studies further examine the available evidence for selected high-ranking candidates.

## 2. Materials and Methods

### 2.1. Dataset Construction and Preprocessing

Association records were obtained from RNADisease v4.0 [3]. RNA names were mapped to stable Ensembl gene identifiers using fixed versions of the HGNC and GENCODE annotations [20,21]. Disease names and identifiers were normalized against Disease Ontology (DO) terms while preserving the disease concepts described in the source records [22]. Reviewed Unified Medical Language System (UMLS) concept identifiers were used when suitable DO mappings were not available [23]. Duplicate records referring to the same normalized gene–disease pair were consolidated, while source records and publication identifiers were retained for traceability. Mappings to external resources used for feature construction were documented separately.

GENCODE Human release 49 on GRCh38.p14 was used to identify lncRNAs meeting the inclusion criteria and select a representative transcript for each gene [21,24]. Transcripts annotated as Ensembl canonical were prioritized. When no canonical transcript was available, a representative transcript was selected using a deterministic ordering based on transcript length and identifier. Selected transcripts were required to be annotated as lncRNAs at both the gene and transcript levels, have a spliced sequence length of at least 200 nucleotides, and match the corresponding reference sequence. Genes whose selected transcripts did not meet these criteria were excluded. The selected sequences were used to generate the sequence and structure features described in Section 2.2.

To compare methods on identical node sets, we retained the intersection of nodes for which the inputs required by all five baseline methods could be constructed under the specified mapping and missing data policies. A symmetric k-core filter was then applied to the retained positive association graph. lncRNA and disease nodes with fewer than k positive associations were removed iteratively until all remaining nodes satisfied the threshold. The main 2-core dataset comprised 1662 lncRNAs, 220 diseases, and 9920 positive associations. Additional datasets with k = 1, 3, and 5 were constructed for sensitivity analyses. The sizes of all four datasets are summarized in Table 1.

### 2.2. Node Content Representations

Each lncRNA was represented by a 702-dimensional vector comprising sequence, structure, and expression features. Sequence embeddings were generated using a pretrained RNA-FM model [25], distributed through MultiMolecule(version 0.2.0) [26], and aggregated across nonoverlapping windows covering the selected transcript sequence to obtain a 640-dimensional vector. Eight structure descriptors were calculated from the analyzed sequence segments and their secondary structures predicted using ViennaRNA (version 2.7.2) [27], summarizing folding energy, nucleotide pairing, sequence composition, and segment length. Expression features were obtained from GTEx profiles across 54 tissue and cell-type categories by matching stable Ensembl gene identifiers [28]. Expression values in transcripts per million (TPM) were transformed using log(1 + TPM). GTEx expression profiles were available for 1657 of the 1662 lncRNAs (99.7%). Missing expression profiles were represented by zero vectors. The sequence, structure, and expression vectors were individually L2-normalized, with zero vectors left unchanged, and concatenated to form the lncRNA representation (640 + 8 + 54). Feature definitions, missing data handling, and extraction settings are provided in Table S1.

Each disease was represented by text, semantic similarity, and graph structure features. Text embeddings were generated using the pretrained S-BioBERT sentence encoder [29,30,31,32]. Disease Ontology-derived text was used for 214 diseases, whereas reviewed UMLS concept labels obtained through NCBI MedGen were used for six diseases without suitable definition text. The resulting 768-dimensional embeddings were L2-normalized, and the encoder parameters remained fixed during association-model training. Semantic similarity and graph structure features were derived from the Medical Subject Headings (MeSH) ontology [33]. Wang semantic similarity, adapted to the MeSH hierarchy, provided a 220-dimensional profile for each disease in the main dataset [34]. Within each fold, similarity columns corresponding to diseases outside the training disease set were set to zero before row-wise L2 normalization. Eight graph structure descriptors were calculated from the MeSH directed acyclic graph and L2-normalized separately. The text, semantic similarity, and graph structure vectors were concatenated to form a 996-dimensional disease representation in the main dataset (768 + 220 + 8). Disease mappings, descriptor definitions, source versions, and extraction settings are provided in Table S1.

### 2.3. Model Architecture and Training

**Figure 1:**
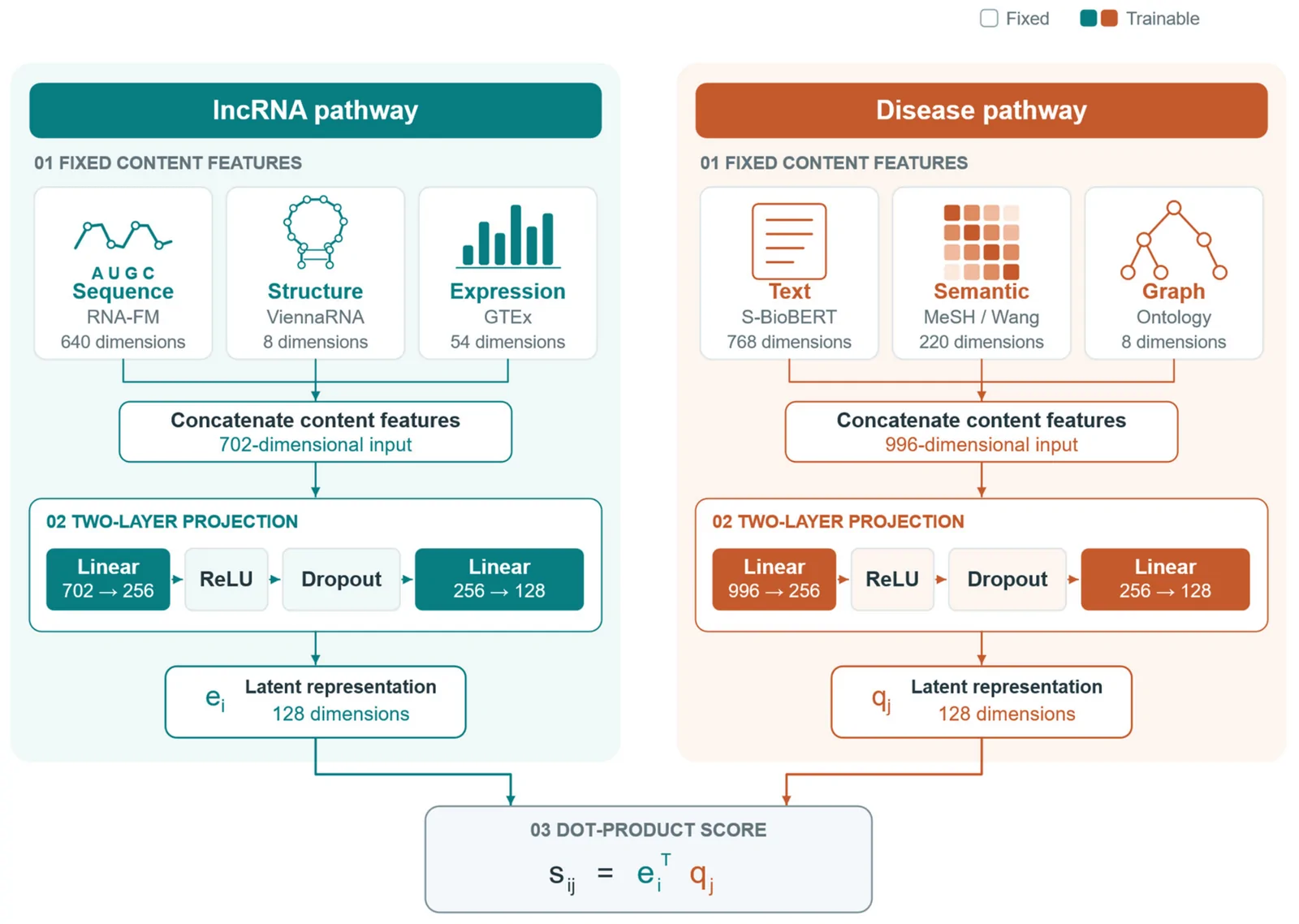
Overview of the CDELDA architecture.

CDELDA maps lncRNA and disease content features into a shared 128-dimensional latent space using separate projection networks (Figure 1). For the main benchmark, the lncRNA and disease inputs contain 702 and 996 dimensions, respectively. Each projection network consists of a linear layer with 256 output dimensions, followed by a rectified linear unit (ReLU), dropout, and a second linear layer that produces a 128-dimensional representation. The two networks have separate trainable parameters. For lncRNA i and disease j, the association logit is computed as the dot product of their projected representations (Equation (1)):

(1) $$z_{ij}=f_{\theta }{\left(x_{i}\right)}^{T}g_{\phi }\left(d_{j}\right)$$

where $x_{i}$ and $d_{j}$ denote the lncRNA and disease content feature vectors, respectively, and $f_{\theta }$ and $g_{\phi }$ denote their corresponding projection networks. The sigmoid-transformed logit is used in the training objective, whereas the logit is used directly to rank candidate associations during evaluation.

The projection networks are trained using a binary cross-entropy objective that assigns equal total weight to positive and eligible unobserved pairs within the training partition (Equation (2)):

(2) $$L=-\frac{1}{2\left|P_{tr}\right|}\sum_{\left(i,j\right)\in P_{tr}}\log\sigma \left(z_{ij}\right)-\frac{1}{2\left|U_{tr}\right|}\sum_{\left(i,j\right)\in U_{tr}}\log\left[1-\sigma \left(z_{ij}\right)\right]$$

where $P_{tr}$ and $U_{tr}$ denote the training positive and eligible unobserved pair sets, respectively, and σ denotes the sigmoid function. Unobserved pairs serve as training controls rather than experimentally confirmed negative associations. Only pairs included in the training mask contribute to the objective. The pretrained encoders remain frozen. The parameters of both projection networks are reinitialized for each training run and optimized using AdamW [35] for 500 parameter updates. Gradients are accumulated across all eligible training pairs before each update. Hyperparameter settings are provided in Supplementary Table S1.

### 2.4. Baseline Methods and Reference Predictors

The primary comparison includes five baseline methods: DSCMF [4], IPCARF [6], SIMCLDA [5], VGAELDA [7], and LDA-VGHB [8]. CDELDA and the baseline methods are evaluated using the same lncRNA and disease sets, positive association labels, training and test partitions, and evaluation candidate masks. Association-derived features are constructed using only the association labels available in the training partition. Method-specific feature inputs, training objectives, and training-pair sampling procedures follow the documented settings of each implementation.

The baseline implementations are derived from the original publications and the available author-released code. DSCMF, IPCARF, SIMCLDA, and LDA-VGHB are designated as reconstructed implementations because their application to the revised benchmark required additional specifications for feature construction, feature integration, or execution settings. VGAELDA uses the author-released model code with reconstructed feature inputs. Procedures not fully specified in the available materials are implemented using fixed, documented settings. Implementation provenance and principal reconstruction choices are summarized in Supplementary Table S2.

Additional reference predictors include CDELDA-DT, Popularity, Random, and matrix factorization (MF). CDELDA-DT was included as a disease-text-only reference variant. It retained the lncRNA inputs and projection architecture of CDELDA but used only the 768-dimensional disease text embeddings as disease inputs. Popularity scores each pair using the product of the lncRNA and disease degrees calculated from positive training associations. Random assigns reproducible random scores. MF represents both node types using trainable node embeddings without content features. Feature sources, preprocessing procedures, hyperparameters, and software versions for the baseline methods and reference predictors are detailed in Supplementary Table S2.

### 2.5. Evaluation Protocols and Association-Label Controls

**Figure 2:**
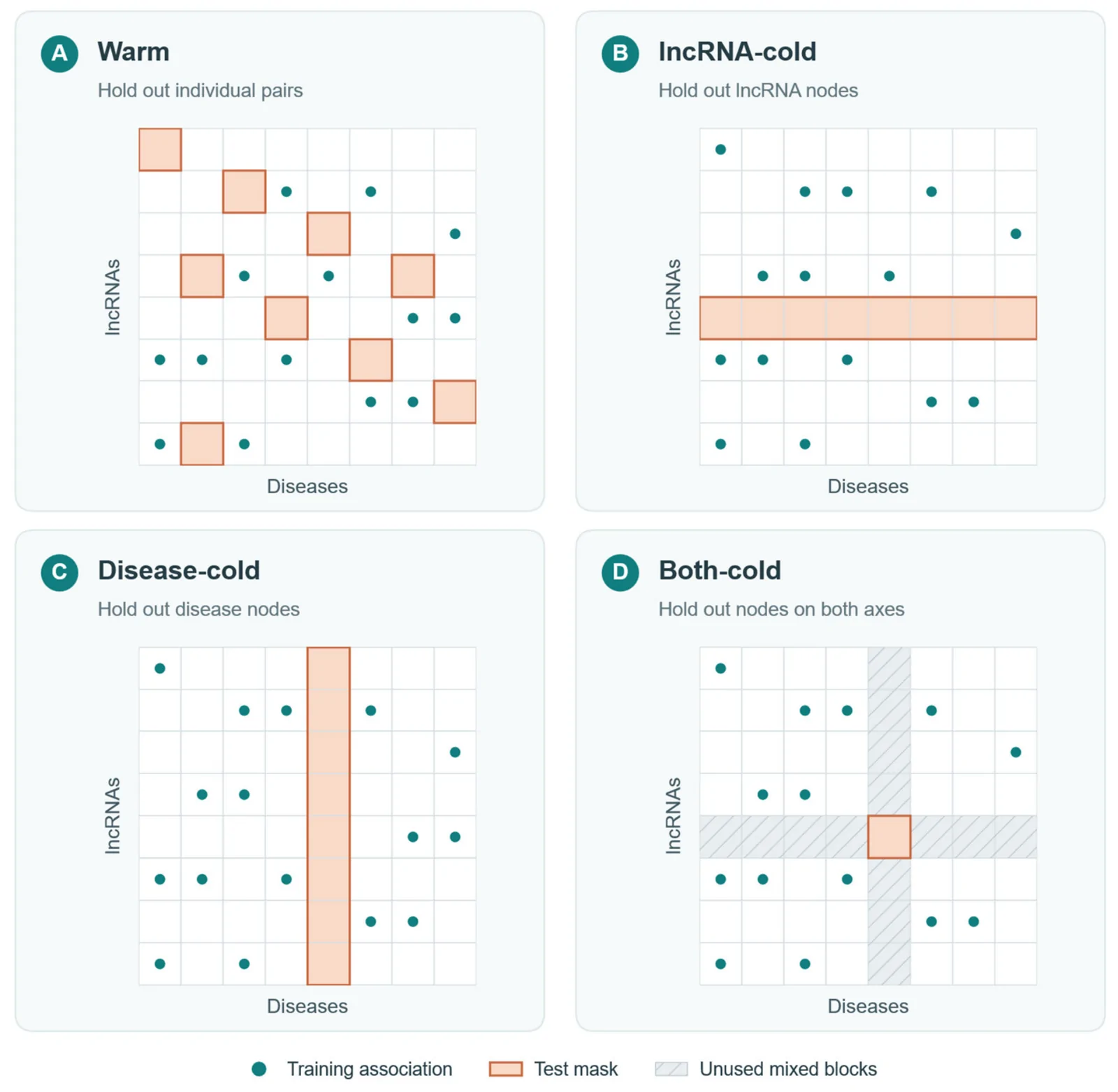
Evaluation protocols: (**A**) warm, (**B**) lncRNA-cold, (**C**) disease-cold, and (**D**) both-cold.

The main comparison uses four evaluation protocols: warm, disease-cold, lncRNA-cold, and both-cold (Figure 2). Each protocol uses five outer folds and five random seeds, yielding 25 evaluations per protocol and 100 evaluations per model. In the warm protocol, positive and eligible unobserved pairs are partitioned separately into five folds. One fold is reserved for testing, and the remaining folds form the training partition. A test pair is retained only if both its lncRNA and disease have at least one positive association in the training partition. Test pairs that do not meet this criterion are excluded from evaluation.

In the disease-cold protocol, all association labels involving the held-out diseases are excluded from training, and evaluation uses pairs between these diseases and the lncRNA set. In the lncRNA-cold protocol, all association labels involving the held-out lncRNAs are excluded from training, and evaluation uses pairs between these lncRNAs and the disease set. In the both-cold protocol, lncRNAs and diseases are each partitioned into five folds, and folds with the same index are held out together. Training uses only pairs between the remaining lncRNAs and diseases, whereas evaluation uses only pairs between the held-out lncRNAs and held-out diseases. Across the cold protocols, association labels involving held-out nodes are excluded from both model training and association-derived feature construction. Labels of test pairs are used only for evaluation. Fold-level test positive counts and protocol-level summaries of the evaluation candidate sets are provided in Table S3.

### 2.6. Evaluation Metrics and Statistical Analysis

The main comparison metric was the disease-macro area under the receiver operating characteristic curve (AUROC). Evaluation used the complete test candidate set, comprising positive associations and unobserved pairs, without subsampling the unobserved pairs. AUROC was calculated separately for each disease with at least one positive association and one unobserved pair. Within each disease, AUROC measures the probability that a randomly selected positive association receives a higher prediction score than a randomly selected unobserved pair, with half credit assigned to tied scores [36]. Disease-specific AUROC values were averaged with equal weights within each fold, followed by averaging across folds within each seed and then across seeds. Each included disease therefore contributed equally to the fold-level metric regardless of its number of positive associations. Disease-macro average precision (AP) was used as a key secondary metric. AP summarizes precision across score thresholds, weighted by the corresponding increases in recall [37]. Precision and recall were additionally calculated for the top 10 and 20 candidates, and normalized discounted cumulative gain (NDCG) was calculated for the top 20 candidates. Precision measures the proportion of selected candidates labeled positive, whereas recall measures the proportion of positive associations in the test set retrieved among the selected candidates. NDCG assigns greater weight to positives appearing earlier in the ranking and normalizes the resulting gain against an ideal ranking [38]. These disease-level metrics used the same disease set and averaging procedure as disease-macro AUROC. Pooled AUROC and AP were also calculated by combining all test pairs within each fold. All metrics were interpreted relative to benchmark labels, because unobserved pairs are not confirmed biological negatives.

To assess sensitivity to the positive-to-unobserved ratio in the main 2-core benchmark, pooled AUROC and AP were additionally calculated for ratios of 1:1, 1:5, and 1:10. All positive associations in the test set were retained, while unobserved pairs were sampled without replacement. Sampling was repeated ten times for each ratio, with the same sampled pairs used across models. Scores were first averaged across sampling repetitions within each fold and then across folds and seeds using the procedure described above. Sampled-control results were reported separately from the full-candidate evaluation.

Exploratory statistical comparisons focused on both-cold evaluation. Conditional uncertainty was estimated using 9999 bootstrap replicates with a product-weight scheme related to crossed-array bootstrapping [39]. Independent unit-mean exponential weights were assigned to lncRNA groups and diseases. The same weights were applied across models, folds, and seeds so that repeated occurrences of each group or disease received a common weight. Within each replicate, disease-level metrics and their averages were recalculated, and paired differences between models were computed using the same test candidates. Approximate 95% confidence intervals were calculated using basic bootstrap intervals. Approximate two-sided *p*-values were obtained from the centered bootstrap distribution of paired differences. Bonferroni correction was applied to the six disease-macro AUROC comparisons of CDELDA against CDELDA-DT and each of the five baseline methods. AP comparisons were treated as secondary analyses. These estimates were conditional on the fitted models and observed benchmark, with predictions held fixed and no retraining performed. They therefore do not quantify the full uncertainty associated with model training or benchmark construction.

### 2.7. Additional Analyses and Case Study

Additional analyses examined the contributions of input features and sensitivity to dataset filtering, as well as the treatment of unobserved pairs. Disease-text embeddings were replaced with zero vectors or permuted separately within the training and test partitions. Feature ablations replaced selected blocks with zeros. Both analyses retained input dimensions and projection architectures and were followed by retraining. For high-confidence exclusion, CDELDA-DT scored unobserved training pairs through three-way pair cross-fitting within each outer training set. The highest-scoring 1% were excluded from the loss without being relabeled as positive, and CDELDA and CDELDA-DT were retrained with the test candidates and labels unchanged. These analyses and the 1-, 3-, and 5-core comparisons used both-cold and disease-cold evaluation with three seeds and five folds per seed, without additional hyperparameter tuning. In a separate analysis using fixed predictions, 0%, 5%, 10%, or 20% of positive associations in the test set were relabeled as unobserved pairs across ten sampling repetitions shared across models. Disease-ranking similarity was evaluated using top-20 overlap, Jaccard similarity, and Spearman rank correlation on common test lncRNA candidates. Disease-input permutation used fitted CDELDA and CDELDA-DT models from both-cold and disease-cold evaluation with five seeds and five folds per seed. Complete disease feature vectors were permuted among held-out diseases within each fold without assigning any disease its own vector. Ten shared permutations were applied across the two models, with model parameters, test candidates, and association labels held fixed.

An illustrative retrospective case study examined breast cancer and prostate cancer using CDELDA predictions from one disease-cold fold. A common lncRNA candidate set was constructed from pairs that were unobserved for both diseases after applying the recorded exclusions. Each lncRNA was included once using its normalized gene identifier. The ten highest-ranked candidates for each disease were selected, and the candidate lists were not revised based on the literature findings. Publications were searched using gene symbols, known aliases, and disease names. Evidence was classified as functional, observational, indirect, or unconfirmed.

## 3. Results

### 3.1. Performance on the Common Benchmark

CDELDA achieved a higher mean disease-macro AUROC than the five literature-based comparators in the lncRNA-cold and both-cold settings (Table 2). In the both-cold setting, its AUROC was 0.6617 ± 0.0312 (mean ± SD), compared with a mean of 0.6123 for SIMCLDA, the highest-scoring literature-based comparator. CDELDA and CDELDA-DT had similar mean AUROCs in the lncRNA-cold setting. In contrast, IPCARF achieved the highest mean AUROC in the warm and disease-cold settings. These results were obtained using the documented implementations or reconstructions on the common benchmark and do not represent the scores originally reported in the corresponding publications.

The relative performance of the methods varied across ranking metrics. SIMCLDA achieved the highest mean disease-macro AP in the both-cold and lncRNA-cold settings, whereas DSCMF and the popularity control achieved the highest values in the disease-cold and warm settings, respectively (Table 3). CDELDA achieved the highest mean precision at 10, recall at 20, and NDCG at 20 in the both-cold setting (Table S4). In the exploratory paired analysis using fixed predictions, the approximate Bonferroni-adjusted intervals for the both-cold AUROC differences between CDELDA and each of the five literature-based comparators were above zero, whereas the interval for the difference from CDELDA-DT included zero (Table S9).

To assess whether the performance difference highlighted in the earlier analysis was consistent across dataset variants, we re-examined the archived comparisons between the LoRA-based CDELDA model and KATZLDA-content. The variants l2d5, l3d5, and l2d10 were constructed by iteratively removing nodes until the minimum numbers of positive associations per lncRNA and per disease reached (2, 5), (3, 5), and (2, 10), respectively. Under both-cold evaluation with a 1:1 positive-to-unobserved ratio, the mean CDELDA-minus-KATZLDA-content AUPR differences were +0.0753 for l2d5, +0.0398 for l3d5, and +0.0142 for l2d10. Recalculation using two-sided paired t-tests on seed-level mean AUPR values from five seeds yielded Bonferroni-adjusted *p*-values of 0.0007, 0.0426, and 0.1785, respectively, with adjustment across the three comparisons. Although all three mean differences were positive, the l2d10 difference was not statistically significant. Thus, the earlier analysis did not establish a statistically significant advantage across all three variants. These historical results clarify the scope of the earlier comparison and are distinguished from the current results, which were obtained using a rebuilt benchmark, a multiview CDELDA model without LoRA, and a different evaluation procedure.

### 3.2. Contribution of Content Features

**Figure 3:**
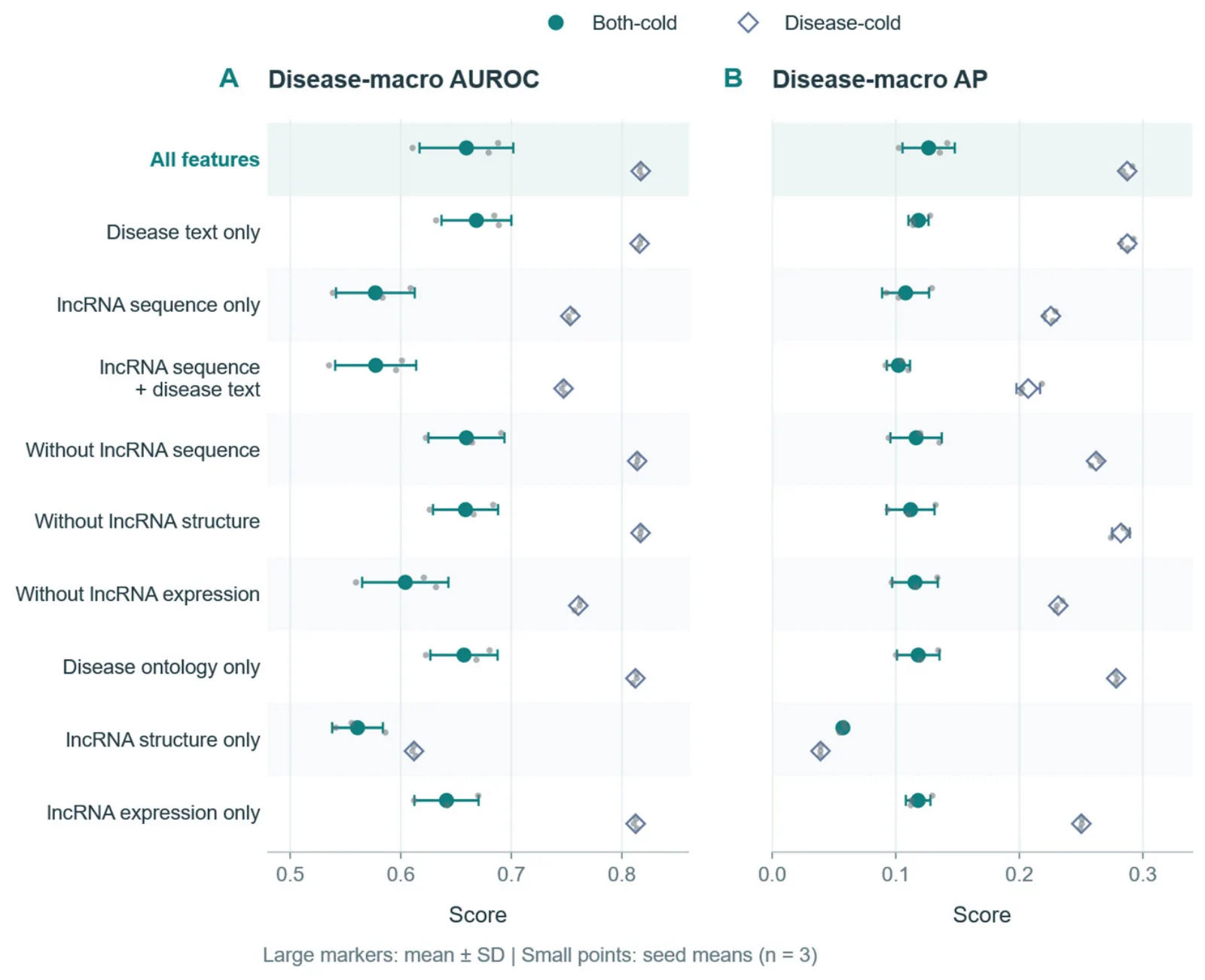
Width-matched feature ablation evaluated by disease-macro AUROC (**A**) and AP (**B**). Teal circles and open blue diamonds denote both-cold and disease-cold results, respectively. Large markers and error bars show the mean ± SD across three seeds. Small gray points show each seed’s five-fold mean.

Feature ablation analyses were performed by setting selected feature blocks to zero while retaining the input dimensions and projection architectures (Figure 3, Table S5). Among the individual lncRNA feature blocks, removing expression features produced the largest decrease in disease-macro AUROC in both-cold and disease-cold evaluations. In both-cold evaluation, disease-macro AUROC decreased from 0.6594 with all features to 0.6041 without expression features. Removing sequence features produced smaller changes in disease-macro AUROC but reduced disease-macro AP in disease-cold evaluation. Removing structural features produced little change in disease-macro AUROC in either setting.

The contribution of disease semantic and graph features varied across evaluation settings and metrics. In both-cold evaluation, the disease-text-only ablation had a higher disease-macro AUROC but a lower disease-macro AP than in the full-feature condition. In disease-cold evaluation, disease-macro AP was similar between these conditions. Thus, retaining all disease feature blocks did not consistently improve both metrics. The disease-text-only ablation retained all lncRNA features and the original disease input dimension, with the semantic and graph feature blocks set to zero. It therefore differed from CDELDA-DT, which used only the 768-dimensional disease text embeddings as disease inputs.

Additional controls were constructed from CDELDA-DT by replacing either the lncRNA or disease content projection network with learnable node embeddings (Table S5). Replacing either network reduced disease-macro AUROC to approximately chance level in both-cold evaluations. In disease-cold evaluation, replacing the disease projection network also resulted in near-chance performance, whereas replacing the lncRNA projection network yielded a smaller decrease relative to CDELDA-DT. In these controls, embeddings for held-out nodes received no association-based training signals. The results therefore characterize the behavior of content-based and node-embedding representations under the specified cold-start settings, rather than providing a parameter-matched estimate of the contribution of content features.

### 3.3. Sensitivity to Dataset and Control Definitions

**Figure 4:**
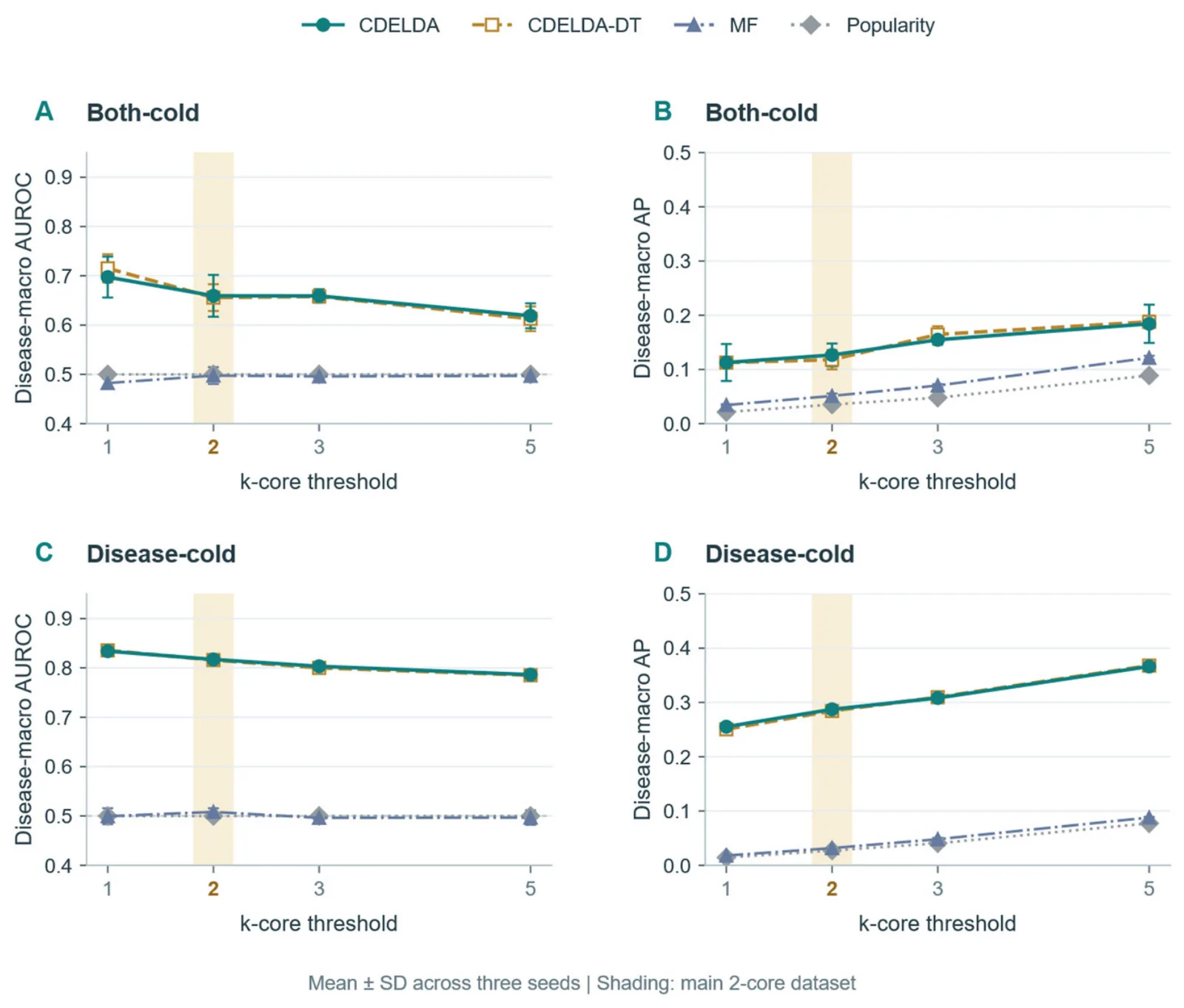
Sensitivity to k-core filtering: (**A**) both-cold disease-macro AUROC, (**B**) both-cold disease-macro AP, (**C**) disease-cold disease-macro AUROC, and (**D**) disease-cold disease-macro AP.

To assess sensitivity to dataset filtering, CDELDA was evaluated on datasets constructed with symmetric k-core thresholds of 1, 2, 3, and 5, using the same three seeds and fixed model settings (Figure 4, Table S6). In both-cold and disease-cold evaluation, higher thresholds were accompanied by lower disease-macro AUROC but higher disease-macro AP. For example, both-cold disease-macro AUROC decreased from 0.6974 at k = 1 to 0.6188 at k = 5. Because k-core filtering changes node composition, connectivity, and the proportion of positive associations together, these changes cannot be attributed to network density alone. To assess sensitivity to training-control selection, an auxiliary procedure using only training data assigned association scores to the unobserved training controls. The top-scoring 1% were excluded from the training controls, and the models were retrained without changing the test labels or candidate sets (Table S7). This exclusion did not consistently improve CDELDA’s disease-macro AUROC or disease-macro AP in both-cold and disease-cold evaluation. Finally, sensitivity to test-control sampling and label completeness was assessed using pooled AP, calculated across all evaluated test pairs rather than averaged across diseases. In both-cold evaluation, CDELDA’s pooled AP decreased from 0.6575 with one sampled unobserved control per positive association to 0.0637 when all test controls were included (Table S7). In a separate analysis, relabeling a subset of observed test positives as unobserved also reduced pooled AP while predictions and candidate sets remained fixed (Table S7). These results show that pooled AP was sensitive to the proportion of labeled positives and the completeness of the observed labels.

### 3.4. Disease-Input Perturbation Analyses

**Figure 5:**
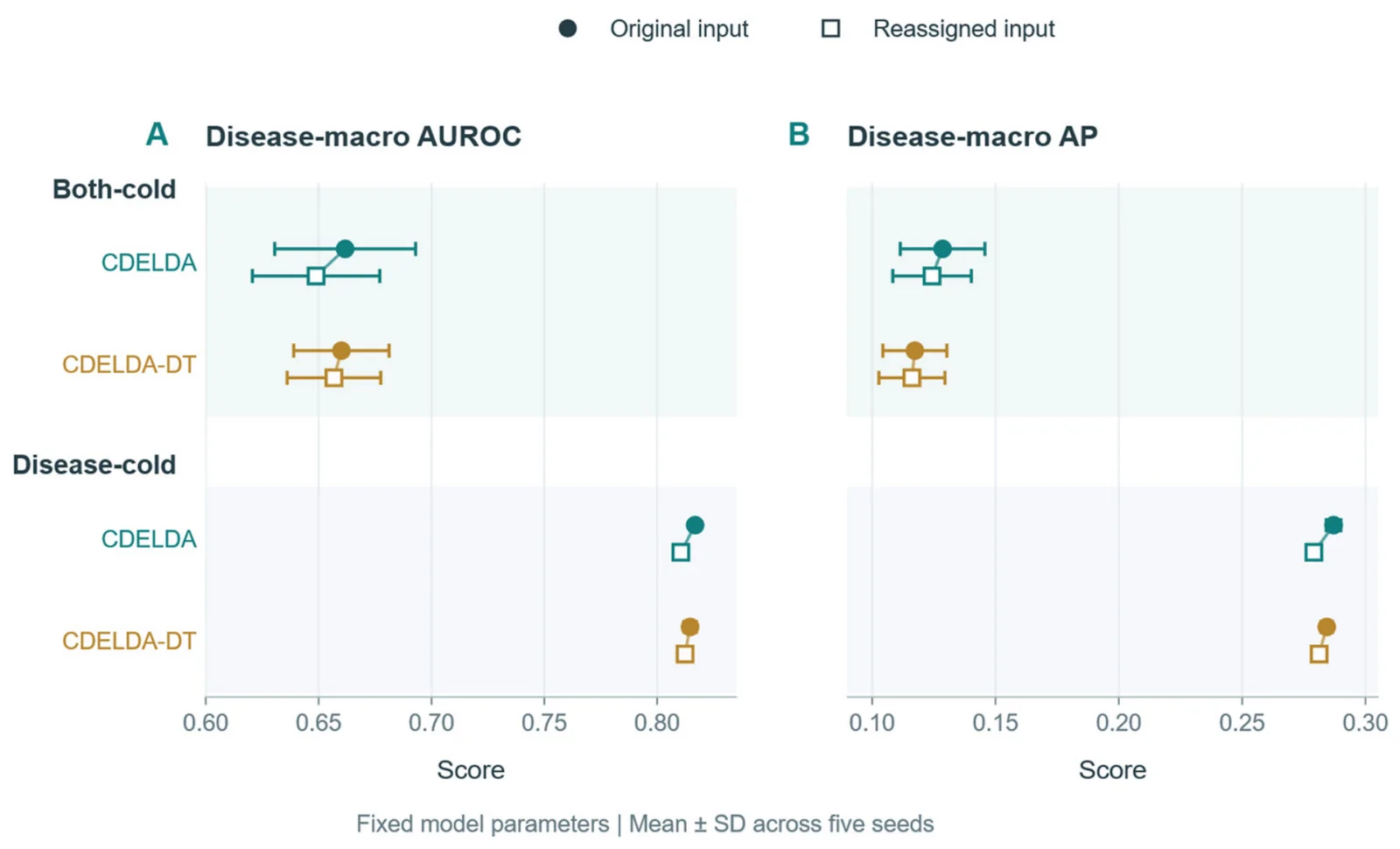
Effect of disease-input reassignment with model parameters fixed.

To examine the role of disease text information, disease text embeddings were either set to zero or randomly reassigned among diseases before model retraining (Table S8). Across three matched seeds, these modifications produced small changes in CDELDA’s disease-macro AUROC in both-cold and disease-cold evaluations. CDELDA-DT also retained ranking performance when its disease text embeddings were set to zero. In this condition, the disease projection network produced a shared representation determined by its learned parameters, including bias terms, rather than disease-specific text representations. A separate analysis assessed the effect of reassigning disease input features after training. Model parameters were held fixed, and input feature vectors were randomly reassigned among held-out diseases without changing the test labels or candidate sets (Figure 5, Table S8). This reassignment reduced CDELDA’s disease-macro AUROC and disease-macro AP in both-cold and disease-cold evaluation. The mean decrease in both-cold disease-macro AUROC was 0.0129. These results indicate that the correspondence between diseases and their input features affected predictive performance, although much of the ranking performance remained after reassignment.

### 3.5. Retrospective Case Studies

Retrospective case studies were conducted for breast cancer and prostate cancer using the ten highest-ranked unobserved lncRNA candidates for each disease from one disease-cold fold. The candidate lists were fixed before the literature review. Among the 20 candidate associations, six had functional evidence, eight had observational evidence, and two had indirect evidence in the reviewed sources. The remaining four associations were classified as unconfirmed (Table 4). MIR210HG ranked first for breast cancer and second for prostate cancer, with functional evidence reported for both associations [40,41,42]. MEG8 ranked third for breast cancer with functional evidence [43] and first for prostate cancer with observational evidence [44]. The similarity of candidate rankings between the two diseases was also assessed (Table S10). The two top-ten lists contained 13 unique lncRNAs, seven of which appeared in both lists. Across the 1227 shared unobserved candidates, prediction scores were highly correlated between breast cancer and prostate cancer (Spearman ρ = 0.955).

## 4. Discussion

Disease-macro AUROC was used as the main comparison metric to assess how well each method distinguished recorded positive associations from unobserved controls within individual diseases. Averaging disease-level AUROC values with equal weight prevents diseases with more evaluated pairs from dominating the overall result. CDELDA showed its clearest relative advantage under both-cold evaluation, although the ranking of methods varied across evaluation settings and metrics. IPCARF achieved the highest disease-macro AUROC in warm and disease-cold evaluation, whereas SIMCLDA achieved the highest disease-macro AP in both-cold and lncRNA-cold evaluation. These differences indicate that within-disease discrimination and the retrieval of positives at higher ranks should be considered together.

An important challenge in practical lncRNA–disease association prediction is to prioritize candidate associations when neither the lncRNA nor the disease has association records in the training data, even though content features are available for both. In this setting, the model cannot rely on association patterns directly observed for either target node. Both-cold evaluation addresses this challenge by excluding association labels involving either test node from training. It therefore complements warm evaluation by assessing prediction for nodes without training associations. CDELDA’s relative advantage in disease-macro AUROC under this condition suggests that its content-based representations were useful for ranking candidate associations in this setting.

Content features allow held-out nodes to be represented without learning node-specific embeddings from their association labels. The ablation experiments examined the contribution of individual feature blocks to predictive performance. Removing expression features produced the largest decrease in disease-macro AUROC among the individual lncRNA feature removals, indicating an important contribution from expression profiles within the present model. Removing sequence or structure features had smaller or metric-dependent effects, and additional disease features did not consistently improve performance over the text-only representation. These results indicate that the predictive contribution of content features depended on the feature block and evaluation setting, rather than increasing consistently with the number of views combined.

The disease-feature perturbation experiments examined how ranking performance depended on the correspondence between diseases and their input features. Reassigning disease feature vectors while keeping the trained model fixed reduced performance, with a mean decrease of 0.0129 in the both-cold disease-macro AUROC. The disease-text-only variant, CDELDA-DT, also retained ranking performance when disease text features were set to zero before retraining. These results show that disrupting the original disease-feature correspondence did not eliminate ranking performance, although the correspondence affected the predictions. Predictive performance alone therefore does not establish that the model provides disease-specific prioritization.

The sensitivity analyses showed that performance should be interpreted alongside the composition of the evaluation data. Pooled AP was lower with the full unobserved candidate set than with balanced controls, illustrating its dependence on positive prevalence [58]. Increasing the k-core threshold was also accompanied by lower AUROC and higher AP in the evaluated cold-start settings. Because k-core filtering changes node composition, connectivity, and positive prevalence simultaneously, this pattern cannot be attributed to association density alone. Unobserved pairs also served as evaluation controls rather than experimentally established negatives. Performance comparisons should therefore use consistent data-construction and evaluation conditions, with the results interpreted in relation to the evaluated candidate set.

The case studies complemented the benchmark evaluation by identifying functional or observational evidence for several highly ranked candidates. Distinguishing these evidence types allowed the predictions to be interpreted in light of the nature of the available support. The strong similarity between the breast and prostate cancer rankings, however, suggests that some candidates were ranked highly across diseases rather than preferentially for a particular disease. The case studies therefore provide retrospective literature support for selected predictions and identify candidates for follow-up, rather than independent validation of the predictions. Disease-specific experiments would help determine the functional relevance of these candidates in the corresponding diseases.

The use of a pretrained text encoder requires a distinction between controlling association labels and excluding prior biological information. Restricting association-derived inputs to training labels does not rule out prior exposure of the encoder to relevant information. The cold-start results should therefore be understood as prediction without training association labels for the held-out nodes, rather than prediction without prior information about them. Overall, CDELDA provides a content-based approach to ranking candidate associations when association records for the target nodes are unavailable during training. Its clearest relative advantage in disease-macro AUROC was observed under both-cold evaluation, suggesting its potential use in prioritizing lncRNA–disease associations for further investigation.

## 5. Conclusions

This study presented CDELDA, a content-based model for prioritizing lncRNA–disease associations, including those involving nodes whose association labels were unavailable during training. A normalized common benchmark enabled comparison with five baseline methods across warm and cold-start evaluation settings. CDELDA showed its clearest relative advantage in disease-macro AUROC under both-cold evaluation, suggesting that content-based representations can support candidate prioritization in this setting. Feature ablation and perturbation analyses characterized the contribution of individual inputs and examined the dependence of ranking performance on disease-feature correspondence. Together, these contributions provide a model and an empirical assessment of content-based prediction for nodes without training associations. Future work should investigate approaches that strengthen disease-specific prioritization and account for uncertainty in unobserved associations. Evaluation on independent datasets with clearly defined temporal cutoffs, together with targeted experimental validation, would help assess the generalizability and biological relevance of the prioritized candidates.

## Figures and Tables

**Table 1:** Dataset sizes after content intersection and symmetric k-core filtering.

k	lncRNAs	Diseases	Positive Associations	Role
1	2722	284	11,042	Sensitivity analysis
2	1662	220	9920	Main benchmark
3	1137	192	8816	Sensitivity analysis
5	596	150	6836	Sensitivity analysis

**Table 2:** Disease-macro AUROC on the common 2-core benchmark.

Method	Warm	lncRNA-Cold	Disease-Cold	Both-Cold
CDELDA	0.8145 ± 0.0021	0.6672 ± 0.0150	0.8168 ± 0.0008	0.6617 ± 0.0312
CDELDA-DT	0.8089 ± 0.0026	0.6672 ± 0.0140	0.8146 ± 0.0028	0.6601 ± 0.0211
DSCMF	0.7086 ± 0.0012	0.5547 ± 0.0077	0.8171 ± 0.0011	0.5460 ± 0.0155
IPCARF	0.8230 ± 0.0023	0.5000 ± 0.0000	0.8234 ± 0.0014	0.5000 ± 0.0000
SIMCLDA	0.7179 ± 0.0063	0.6053 ± 0.0142	0.4704 ± 0.0313	0.6123 ± 0.0221
VGAELDA	0.7967 ± 0.0024	0.5301 ± 0.0224	0.5440 ± 0.0041	0.5000 ± 0.0000
LDA-VGHB	0.8104 ± 0.0035	0.5088 ± 0.0214	0.7924 ± 0.0113	0.5174 ± 0.0308
MF	0.6330 ± 0.0112	0.4992 ± 0.0104	0.5058 ± 0.0151	0.4994 ± 0.0175
Popularity	0.8147 ± 0.0039	0.5000 ± 0.0000	0.5000 ± 0.0000	0.5000 ± 0.0000
Random	0.4984 ± 0.0039	0.4945 ± 0.0034	0.4924 ± 0.0049	0.5010 ± 0.0197

**Table 3:** Disease-macro average precision on the common 2-core benchmark.

Method	Warm	lncRNA-Cold	Disease-Cold	Both-Cold
CDELDA	0.3752 ± 0.0038	0.1227 ± 0.0215	0.2871 ± 0.0030	0.1285 ± 0.0172
CDELDA-DT	0.3720 ± 0.0054	0.1302 ± 0.0285	0.2843 ± 0.0024	0.1173 ± 0.0130
DSCMF	0.3004 ± 0.0013	0.0605 ± 0.0016	0.3022 ± 0.0006	0.0536 ± 0.0047
IPCARF	0.3744 ± 0.0076	0.0354 ± 0.0005	0.2931 ± 0.0025	0.0352 ± 0.0012
SIMCLDA	0.2163 ± 0.0043	0.1315 ± 0.0221	0.0623 ± 0.0083	0.1348 ± 0.0252
VGAELDA	0.3576 ± 0.0068	0.0552 ± 0.0026	0.0634 ± 0.0050	0.0352 ± 0.0012
LDA-VGHB	0.3444 ± 0.0050	0.0519 ± 0.0026	0.1463 ± 0.0301	0.0531 ± 0.0017
MF	0.0920 ± 0.0043	0.0493 ± 0.0016	0.0322 ± 0.0010	0.0513 ± 0.0051
Popularity	0.3871 ± 0.0043	0.0354 ± 0.0005	0.0273 ± 0.0000	0.0352 ± 0.0012
Random	0.0513 ± 0.0019	0.0515 ± 0.0009	0.0315 ± 0.0008	0.0502 ± 0.0034

**Table 4:** The literature evidence for the ten highest-ranked unobserved lncRNA candidates in breast cancer and prostate cancer.

**A. Breast Cancer**			
**Rank**	**lncRNA**	**Evidence Category**	**Source Identifier**
1	MIR210HG	Functional	PMID: 35346372 [40], 31399552 [41]
2	ESRG	Observational	PMID: 39310490 [45]
3	MEG8	Functional	PMID: 39706842 [43]
4	LINC02562	Observational	PMID: 42066072 [46]
5	NUTM2A-AS1	Unconfirmed	—
6	DNM3OS	Unconfirmed	—
7	DLEU2	Functional	PMID: 38296962 [47]
8	FAM30A	Observational	PMID: 41670092 [48]
9	SLCO4A1-AS1	Observational	PMID: 37726723 [49]
10	GATA6-AS1	Observational	PMID: 29123410 [50], 29795261 [51]
**B. Prostate Cancer**			
**Rank**	**lncRNA**	**Evidence Category**	**Source Identifier**
1	MEG8	Observational	PMID: 27634882 [44]
2	MIR210HG	Functional	PMID: 37758909 [42]
3	ESRG	Unconfirmed	—
4	LINC02562	Indirect	PMID: 40530115 [52]
5	DNM3OS	Indirect	PMID: 31694982 [53]
6	NUTM2A-AS1	Functional	PMID: 38583010 [54]
7	GATA6-AS1	Observational	DOI:10.3390/informatics11020014 [55]
8	SNHG18	Functional	PMID: 41702447 [56]
9	LUADT1	Unconfirmed	—
10	MIR100HG	Observational	PMID: 36911392 [57]

## Data Availability

Source association records were obtained from RNADisease v4.0 (https://www.rnadisease.org/, accessed on 16 September 2026). Code and numerical result aggregates for the revised benchmark and implementation are available at https://github.com/rlatkddnr122/CDELDA (accessed on 16 September 2026), in the reproducibility/20260910 directory. Required external inputs and their access and redistribution restrictions are documented in the repository.

## Supplementary Materials

The following supporting information can be downloaded at: https://www.mdpi.com/article/10.3390/biomedicines14092121/s1. The supporting information accompanying this manuscript contains Tables S1–S10. Table S1. Feature extraction and training settings. Table S2. Baseline implementation and reconstruction settings. Table S3. Test support by protocol and fold. Table S4. Additional ranking and pooled metrics. Table S5. Content ablation and node-embedding controls. Table S6. Sensitivity to k-core filtering. Table S7. Sensitivity to unobserved-control definitions. Table S8. Disease-input perturbations and ranking similarity. Table S9. Exploratory paired comparisons in both-cold evaluation. Table S10. Candidate-level literature assessments.

## Abbreviations

AP	Average precision
AUROC	Area under the receiver operating characteristic curve
DO	Disease Ontology
GTEx	Genotype-Tissue Expression
HGNC	HUGO Gene Nomenclature Committee
LDA	Long non-coding RNA–disease association
lncRNA	Long non-coding RNA
MeSH	Medical Subject Headings
MF	Matrix factorization
NDCG	Normalized discounted cumulative gain
ReLU	Rectified linear unit
