# Supplementary material for: CDELDA: A Content-Based Dual-Encoder for Cold-Start lncRNA–Disease Association Prediction
Source: Biomedicines. 2026 Sep 19;14(9):2121. doi: 10.3390/biomedicines14092121 (PMC13604804; doi:10.3390/biomedicines14092121)
Supplement: Supplementary file 1 [file biomedicines-14-02121-s001.zip › biomedicines-4557477-supplementary.pdf]

biomedicines

Supplementary Materials

CDELDA: A Content-Based Dual-Encoder for Cold-Start lncRNA–Disease Association Prediction

Sanguk Kim, Taehyeon Yun and Jihwan Ha

Tables S1–S10 provide implementation settings, evaluation support, additional results and candidate-level evidence. Dataset construction and primary AUROC/AP comparisons are reported in main Table 1 and Tables 2–3. Resource and method references are given in the main manuscript. Source identifiers in Table S10 link directly to the assessed literature.

Table S1. Feature extraction and training settings.

Category	Input or setting	Width	Construction or settings
External content	RNA-FM	640	All nonoverlapping windows of at most 1,022 nt cover the representative GENCODE 49 transcript. Aggregate nucleotide tokens only, with length-weighted whole-transcript pooling.
External content	ViennaRNA	8	Analyze the first at most 1,500 nt. Descriptors: MFE/nt, paired fraction, GC fraction, paired/unpaired transition frequency, normalized mean paired and unpaired run lengths, bracket imbalance/nt, and analyzed length/1,500.
External content	GTEx	54	log(1 + TPM) over 54 tissue and cell-type categories. Profiles exist for 1,657/1,662 lncRNAs. Five missing rows are zero-filled and distinguished from observed all-zero profiles in metadata, without an input missingness indicator.
External content	S-BioBERT	768	SentenceTransformer output with saved pooling settings and at most 75 tokens including special tokens. DO-derived text for 214 diseases and reviewed UMLS concept labels through NCBI MedGen for six diseases.
External content	MeSH semantic profile	220	MeSH 2026, Wang attenuation 0.5. The 220 disease nodes map to 206 MeSH proxies without merging disease labels. Set columns outside the training disease set to zero before row normalization.
External content	MeSH graph	8	Shortest root depth/20, ancestor count/50, descendant count/100, parent count/5, child count/20, descendant leaf count/100, Seco information content, and leaf indicator. Information content = 1 − log(descendants + 1)/log(31,110).
External content	BioVec	300	Three 100-dimensional frame-sum vectors trained on the selected sequence corpus without association labels. All 1,662 rows are nonzero. Fixed reconstruction settings are listed in Table S2.
External content	Disease GO profile	17,565	Union of directly exported GO annotations for retained disease-associated genes. All 220 profiles are nonempty. GO Jaccard similarities are available for every disease pair.
External content	Disease–gene matrix	17,039	DisGeNET v7 with reviewed CUI bindings and target-gene exclusion. The 220 × 17,039 binary matrix has 226,860 links and no empty disease rows. Used by VGAELDA.
Model fitting	lncRNA projection	—	702 → 256 → ReLU → Dropout(0.2) → 128
Model fitting	Disease projection	—	CDELDA: 996 → 256 → ReLU → Dropout(0.2) → 128. CDELDA-DT: 768 → 256 → ReLU → Dropout(0.2) → 128.
Model fitting	Score	—	Raw dot product of the two 128-dimensional representations. No output L2 normalization.
Model fitting	Objective	—	0.5 × mean positive-pair binary cross-entropy + 0.5 × mean unobserved-control binary cross-entropy, within the training mask.

Category	Input or setting	Width	Construction or settings
Model fitting	Optimizer	—	AdamW. Learning rate 0.001, betas (0.9, 0.999), epsilon 1e−8. Weight decay 0.001 on non-bias weights and zero on biases.
Model fitting	Training budget	—	500 parameter updates. Final checkpoint, without early stopping. Gradients accumulated over the full training objective using disease chunks of 16.
Model fitting	Replicates	—	Five seeds (41001–41005), five folds per seed for main experiments. Three seeds (41001–41003) for matched feature and sensitivity analyses.
Model fitting	Determinism	—	Deterministic PyTorch algorithms, TF32 disabled, four PyTorch CPU threads.
Model fitting	RNA-FM checkpoint	—	7d6e73ad3b48e042b378f9a788a56ccb4d573a27
Model fitting	S-BioBERT checkpoint	—	21e0a553d5ef08802bacbd8331dfb93b5299c151

Sequence, structure and expression blocks are separately row-wise L2-normalized and concatenated (702 dimensions). Disease text, semantic and graph blocks are separately normalized and concatenated (996 dimensions at k = 2). Zero rows remain zero. All sequence/text encoders remain frozen. Ontology features use external MeSH structure, not held-out association labels. Label separation cannot establish absence of pretraining exposure.

Checkpoint revisions refer to multimolecule/rnafm and pritamdeka/S-BioBert-snli-multinli-stsb, respectively. Each run starts with newly initialized projection parameters. Fixed external content remains available for held-out nodes, whereas association-derived features and the fitting objective use training labels only.

The four protocols use common masks across all ten main methods. When lncRNAs are held out, inherited identifier-risk links and exact sequence-hash matches define 1,660 groups among 1,662 genes, including two groups of two. Whole groups are assigned with seeded node-count balancing. This is not a general sequence-similarity threshold and does not merge gene identities. Both-cold evaluates only five same-index held-out blocks per seed, not all 25 cross-combinations. Mixed training/held-out blocks are unused in that split.

Within each fold, disease-level metrics average diseases with positive and unobserved test support. Five folds are averaged within each seed. Unless stated otherwise, values are mean ± sample standard deviation across five seed means (41001–41005), not across 25 independent folds. Three-seed analyses use 41001–41003. Pooled metrics combine test pairs within each fold and are distinct from disease-macro metrics. Unobserved pairs are operational controls, not confirmed biological negatives.

Table S2. Baseline implementation and reconstruction settings.

Predictor class	Method	Implementation and settings
Literature baseline	DSCMF*	Retains the unweighted source factorization core, numerically compared with the Octave source. The missing driver uses WKNKN (K = 5, η = 0.7), training-mask projection, SVD initialization and a masked solver. Fixed settings: rank 64, λh = 0.5, λlnc = λdisease = 0.01, 100 sweeps. All-zero neighbor weights produce zero directional completion. Nonzero-weight arithmetic is retained without an added epsilon.
Literature baseline	IPCARF*	Retains the lncRNA GIP path, incremental PCA and random forest. Disease similarities are (semantic-1 + semantic-2 + GIP)/3. Pair-vector ordering, semantic-2 background and integration are reconstruction choices. IPCA fits training pairs only, with 128 components and batch size 1,024. Forest: 1,500 trees, Gini, square-root feature sampling, unlimited depth, minimum split 2, minimum leaf 1, bootstrap enabled, 1:1 training controls.
Literature baseline	SIMCLDA*	Retains the source neighbor threshold, sequential cold-profile completion and SIMC λ = 1. Dimensionality thresholds 0.8/0.6 use cumulative singular-value sums, not centered-PCA explained variance. Maximum 1,000 proximal iterations, relative-objective tolerance 1e−5. Unavailable sequence-similarity settings are replaced with normalized Smith–Waterman scores: match +2, mismatch −1, gap-open 3, extension 1. These alignment settings are not attributed to the original authors.

Predictor class	Method	Implementation and settings
Literature baseline	VGAELDA†	Uses public model code with rebuilt common-node inputs. BioVec: 300 dimensions, three frames, selected sequence corpus, skip-gram, window 25, minimum count 1, seed 1, five epochs, one worker. Unpublished author preprocessing settings were not recovered. Inputs use column-wise min–max scaling. Reciprocal 10-neighbor covariate graphs include self-loops. Hidden width 256, 500 alternating epochs, Adam learning rate 0.01, weight decay 1e–5, $\alpha = 0.5$.
Literature baseline	LDA-VGHB*	Retains source VGAE (100 → 5, 250 epochs), training-only SVD (rank 5, at most 200 iterations) and BoostingMachine. VGAE: learning rate 0.001, dropout 0, positive weight 5, normalization 0.1. Fusion $\alpha = 0.5$ with a zero-support fallback. Boosting: 1,000 rounds, learning rate 0.001, depth 1–25, subsampling 0.8, 1:1 controls. Python 3.7, TensorFlow 2.6.0 compatibility-v1, NumPy 1.19.5, SciPy 1.6.3, scikit-learn 0.24.2. Official IBM Pai4sk 1.6.0 replaces unavailable README version 3.5.2. Original-backend equivalence is not claimed.
Reference predictor	CDELDA-DT	Disease-text-only reference with the full lncRNA input and a 768 → 256 → 128 disease projection. Other training settings match CDELDA (Table S1).
Reference predictor	Matrix factorization (MF)	128-dimensional learnable node embeddings on both axes. Raw dot-product scoring and the same balanced objective, AdamW settings and 500 updates as CDELDA.
Reference predictor	Popularity	Product of the lncRNA and disease positive degrees computed from the training association matrix.
Reference predictor	Random	Reproducible uniformly distributed scores in [0, 1) from a seeded NumPy random stream.

* Reconstructed implementations. † Author-released model code with reconstructed external inputs. All are evaluated on the common revised benchmark, not claimed as reproductions of the original published scores. Association-derived similarities and fitting inputs use training labels only. Differences in preprocessing, external inputs and fixed hyperparameters remain part of these method comparisons.

Table S3. Test support by protocol and fold.

Support measure	Seed	Fold	Warm	lncRNA-cold	Disease-cold	Both-cold
Test positives	41001	0	1,939	2,178	2,755	622
Test positives	41001	1	1,935	2,084	2,423	511
Test positives	41001	2	1,934	1,752	879	154
Test positives	41001	3	1,927	1,891	2,089	425
Test positives	41001	4	1,926	2,015	1,774	354
Test positives	41002	0	1,929	1,672	1,729	279
Test positives	41002	1	1,929	1,931	1,705	363
Test positives	41002	2	1,923	1,816	1,594	293
Test positives	41002	3	1,936	2,173	3,412	728
Test positives	41002	4	1,938	2,328	1,480	393
Test positives	41003	0	1,911	2,113	2,233	456
Test positives	41003	1	1,919	1,874	1,921	340
Test positives	41003	2	1,928	1,931	2,000	401
Test positives	41003	3	1,943	1,827	2,371	425
Test positives	41003	4	1,928	2,175	1,395	282
Test positives	41004	0	1,919	2,181	2,421	514

Support measure	Seed	Fold	Warm	lncRNA-cold	Disease-cold	Both-cold
Test positives	41004	1	1,930	1,837	2,174	403
Test positives	41004	2	1,927	1,895	1,874	340
Test positives	41004	3	1,932	1,951	867	178
Test positives	41004	4	1,936	2,056	2,584	540
Test positives	41005	0	1,924	2,148	1,865	405
Test positives	41005	1	1,928	2,012	1,848	377
Test positives	41005	2	1,926	1,906	2,854	554
Test positives	41005	3	1,936	1,988	1,735	349
Test positives	41005	4	1,942	1,866	1,618	293
Test positives (range)	All	All	1,911–1,943	1,672–2,328	867–3,412	154–728
Unobserved controls (range)	All	All	68,211–70,066	70,547–71,393	69,396–72,019	13,858–14,457
All disease queries (range)	All	All	215–220	220–220	44–44	44–44
AUROC/AP queries (range)	All	All	157–181	140–205	44–44	25–41
Candidates per query (range)	All	All	244–389	272–333	1,414–1,662	275–333

Fold indices are zero-based. Test-positive counts are shown for each protocol, seed and fold. Rows labeled "All" show minima–maxima over the 25 seed-fold evaluations per protocol. All ten methods share the same test masks. Controls exclude known positives and documented exclusion pairs. AUROC/AP queries require both positive and unobserved support. Both-cold does not test every possible pair within a seed (Table S1).

Table S4. Additional ranking and pooled metrics.

Protocol	Method	Precision at 10	Recall at 10	Precision at 20	Recall at 20	NDCG at 20	Pooled AUROC	Pooled AP
Warm	CDELDA	0.2296 ± 0.0044	0.4357 ± 0.0064	0.1648 ± 0.0026	0.5263 ± 0.0039	0.4837 ± 0.0039	0.9371 ± 0.0003	0.4182 ± 0.0054
Warm	CDELDA-DT	0.2293 ± 0.0054	0.4288 ± 0.0060	0.1633 ± 0.0030	0.5174 ± 0.0046	0.4795 ± 0.0051	0.9363 ± 0.0004	0.4177 ± 0.0034
Warm	DSCMF*	0.1871 ± 0.0052	0.3529 ± 0.0030	0.1392 ± 0.0028	0.4294 ± 0.0029	0.3921 ± 0.0015	0.8515 ± 0.0027	0.3161 ± 0.0021
Warm	IPCARF*	0.2405 ± 0.0036	0.4452 ± 0.0032	0.1768 ± 0.0036	0.5400 ± 0.0054	0.4827 ± 0.0088	0.9093 ± 0.0028	0.3732 ± 0.0039
Warm	SIMCLDA*	0.1246 ± 0.0059	0.3046 ± 0.0109	0.0944 ± 0.0021	0.3843 ± 0.0067	0.2875 ± 0.0034	0.8263 ± 0.0017	0.1088 ± 0.0016
Warm	VGAELDA†	0.2187 ± 0.0026	0.4091 ± 0.0078	0.1540 ± 0.0021	0.4979 ± 0.0055	0.4608 ± 0.0050	0.9124 ± 0.0008	0.3798 ± 0.0028
Warm	LDA-VGHB*	0.2298 ± 0.0057	0.4039 ± 0.0069	0.1662 ± 0.0040	0.4991 ± 0.0069	0.4503 ± 0.0076	0.9079 ± 0.0013	0.3676 ± 0.0038
Warm	MF	0.0645 ± 0.0049	0.1086 ± 0.0093	0.0568 ± 0.0024	0.1739 ± 0.0102	0.1213 ± 0.0076	0.7003 ± 0.0041	0.0727 ± 0.0047
Warm	Popularity	0.2477 ± 0.0039	0.4399 ± 0.0039	0.1780 ± 0.0031	0.5346 ± 0.0073	0.5026 ± 0.0066	0.9320 ± 0.0005	0.4223 ± 0.0013

Protocol	Method	Precision at 10	Recall at 10	Precision at 20	Recall at 20	NDCG at 20	Pooled AUROC	Pooled AP
Warm	Random	0.0373 ± 0.0025	0.0306 ± 0.0043	0.0355 ± 0.0012	0.0596 ± 0.0034	0.0517 ± 0.0029	0.4999 ± 0.0019	0.0273 ± 0.0002
lncRNA-cold	CDELDA	0.0940 ± 0.0064	0.1411 ± 0.0215	0.0797 ± 0.0066	0.2282 ± 0.0382	0.1716 ± 0.0290	0.9019 ± 0.0016	0.2834 ± 0.0082
lncRNA-cold	CDELDA-DT	0.1018 ± 0.0080	0.1623 ± 0.0264	0.0805 ± 0.0042	0.2266 ± 0.0394	0.1792 ± 0.0353	0.9030 ± 0.0013	0.2842 ± 0.0053
lncRNA-cold	DSCMF*	0.0455 ± 0.0020	0.0376 ± 0.0036	0.0451 ± 0.0016	0.0742 ± 0.0067	0.0644 ± 0.0033	0.8268 ± 0.0019	0.2567 ± 0.0012
lncRNA-cold	IPCARF*	0.0386 ± 0.0070	0.0325 ± 0.0114	0.0372 ± 0.0043	0.0599 ± 0.0138	0.0535 ± 0.0098	0.8337 ± 0.0083	0.1587 ± 0.0172
lncRNA-cold	SIMCLDA*	0.0807 ± 0.0035	0.1409 ± 0.0168	0.0598 ± 0.0041	0.1811 ± 0.0295	0.1703 ± 0.0256	0.6693 ± 0.0143	0.0679 ± 0.0019
lncRNA-cold	VGAELDA†	0.0420 ± 0.0045	0.0398 ± 0.0162	0.0440 ± 0.0060	0.0915 ± 0.0326	0.0677 ± 0.0106	0.8549 ± 0.0028	0.1855 ± 0.0033
lncRNA-cold	LDA-VGHB*	0.0354 ± 0.0019	0.0308 ± 0.0049	0.0353 ± 0.0017	0.0626 ± 0.0092	0.0519 ± 0.0051	0.7279 ± 0.0425	0.0668 ± 0.0151
lncRNA-cold	MF	0.0353 ± 0.0015	0.0262 ± 0.0014	0.0344 ± 0.0007	0.0526 ± 0.0025	0.0472 ± 0.0008	0.5013 ± 0.0051	0.0278 ± 0.0004
lncRNA-cold	Popularity	0.0386 ± 0.0070	0.0325 ± 0.0114	0.0372 ± 0.0043	0.0599 ± 0.0138	0.0535 ± 0.0098	0.5000 ± 0.0000	0.0272 ± 0.0000
lncRNA-cold	Random	0.0372 ± 0.0028	0.0306 ± 0.0050	0.0358 ± 0.0013	0.0568 ± 0.0028	0.0517 ± 0.0021	0.4996 ± 0.0018	0.0274 ± 0.0002
Disease-cold	CDELDA	0.3997 ± 0.0023	0.3121 ± 0.0018	0.2812 ± 0.0033	0.3681 ± 0.0032	0.4730 ± 0.0014	0.7328 ± 0.0128	0.1390 ± 0.0142
Disease-cold	CDELDA-DT	0.3999 ± 0.0022	0.3111 ± 0.0026	0.2790 ± 0.0010	0.3660 ± 0.0034	0.4695 ± 0.0024	0.7201 ± 0.0151	0.1516 ± 0.0099
Disease-cold	DSCMF*	0.3996 ± 0.0016	0.3138 ± 0.0016	0.2909 ± 0.0020	0.3731 ± 0.0021	0.4899 ± 0.0018	0.7331 ± 0.0059	0.1489 ± 0.0043
Disease-cold	IPCARF*	0.3969 ± 0.0016	0.3092 ± 0.0021	0.2886 ± 0.0002	0.3752 ± 0.0014	0.4779 ± 0.0037	0.7506 ± 0.0073	0.1497 ± 0.0032
Disease-cold	SIMCLDA*	0.0913 ± 0.0119	0.0510 ± 0.0134	0.0819 ± 0.0103	0.0762 ± 0.0186	0.1094 ± 0.0169	0.6913 ± 0.0213	0.0708 ± 0.0045
Disease-cold	VGAELDA†	0.0785 ± 0.0105	0.0440 ± 0.0024	0.0672 ± 0.0101	0.0565 ± 0.0051	0.0940 ± 0.0114	0.4823 ± 0.0229	0.0427 ± 0.0040
Disease-cold	LDA-VGHB*	0.2003 ± 0.0467	0.1272 ± 0.0527	0.1735 ± 0.0339	0.1956 ± 0.0738	0.2460 ± 0.0582	0.6733 ± 0.0103	0.0810 ± 0.0109
Disease-cold	MF	0.0307 ± 0.0018	0.0087 ± 0.0030	0.0310 ± 0.0019	0.0164 ± 0.0031	0.0341 ± 0.0010	0.5030 ± 0.0097	0.0276 ± 0.0010
Disease-cold	Popularity	0.0265 ± 0.0069	0.0062 ± 0.0024	0.0288 ± 0.0084	0.0135 ± 0.0060	0.0313 ± 0.0090	0.5000 ± 0.0000	0.0272 ± 0.0000
Disease-cold	Random	0.0289 ± 0.0034	0.0059 ± 0.0012	0.0292 ± 0.0025	0.0112 ± 0.0014	0.0312 ± 0.0030	0.5000 ± 0.0030	0.0274 ± 0.0002
Both-cold	CDELDA	0.0981 ± 0.0126	0.1584 ± 0.0500	0.0784 ± 0.0067	0.2247 ± 0.0548	0.1782 ± 0.0282	0.6631 ± 0.0133	0.0637 ± 0.0042
Both-cold	CDELDA-DT	0.0951 ± 0.0112	0.1653 ± 0.0459	0.0779 ± 0.0037	0.2246 ± 0.0437	0.1660 ± 0.0207	0.6513 ± 0.0172	0.0677 ± 0.0086

Protocol	Method	Precision at 10	Recall at 10	Precision at 20	Recall at 20	NDCG at 20	Pooled AUROC	Pooled AP
Both-cold	DSCMF*	0.0364 ± 0.0043	0.0194 ± 0.0053	0.0450 ± 0.0057	0.0545 ± 0.0143	0.0526 ± 0.0092	0.6118 ± 0.0038	0.0491 ± 0.0031
Both-cold	IPCARF*	0.0365 ± 0.0053	0.0304 ± 0.0079	0.0354 ± 0.0041	0.0544 ± 0.0169	0.0498 ± 0.0056	0.6441 ± 0.0192	0.0610 ± 0.0085
Both-cold	SIMCLDA*	0.0793 ± 0.0129	0.1427 ± 0.0431	0.0600 ± 0.0065	0.1840 ± 0.0514	0.1698 ± 0.0365	0.6436 ± 0.0226	0.0667 ± 0.0045
Both-cold	VGAELDA†	0.0365 ± 0.0053	0.0304 ± 0.0079	0.0354 ± 0.0041	0.0544 ± 0.0169	0.0498 ± 0.0056	0.5000 ± 0.0000	0.0273 ± 0.0009
Both-cold	LDA-VGHB*	0.0383 ± 0.0069	0.0294 ± 0.0098	0.0390 ± 0.0040	0.0662 ± 0.0162	0.0563 ± 0.0060	0.5002 ± 0.0250	0.0285 ± 0.0034
Both-cold	MF	0.0353 ± 0.0040	0.0293 ± 0.0141	0.0344 ± 0.0022	0.0522 ± 0.0109	0.0483 ± 0.0076	0.5041 ± 0.0060	0.0280 ± 0.0011
Both-cold	Popularity	0.0365 ± 0.0053	0.0304 ± 0.0079	0.0354 ± 0.0041	0.0544 ± 0.0169	0.0498 ± 0.0056	0.5000 ± 0.0000	0.0273 ± 0.0009
Both-cold	Random	0.0381 ± 0.0040	0.0303 ± 0.0066	0.0360 ± 0.0022	0.0615 ± 0.0156	0.0528 ± 0.0066	0.4996 ± 0.0089	0.0280 ± 0.0008

Five seeds. Each cell shows the mean on the first line and ± sample SD on the second. Ranking metrics use supported disease queries, whereas pooled AUROC/AP combine all test pairs within each fold. Primary disease-macro AUROC and AP are in main Tables 2 and 3. Method reconstruction symbols are defined in Table S2.

Table S5. Content ablation and node-embedding controls.

Analysis	Model or feature configuration	Seeds	Both-cold AUROC	Both-cold AP	Disease-cold AUROC	Disease-cold AP
Feature ablation	All features	3	0.6594 ± 0.0424	0.1266 ± 0.0211	0.8171 ± 0.0010	0.2873 ± 0.0038
Feature ablation	Disease text only	3	0.6685 ± 0.0316	0.1184 ± 0.0081	0.8161 ± 0.0019	0.2874 ± 0.0050
Feature ablation	lncRNA sequence only	3	0.5770 ± 0.0356	0.1080 ± 0.0190	0.7534 ± 0.0024	0.2255 ± 0.0047
Feature ablation	lncRNA sequence + disease text	3	0.5773 ± 0.0366	0.1021 ± 0.0094	0.7473 ± 0.0015	0.2072 ± 0.0096
Feature ablation	Without lncRNA sequence	3	0.6594 ± 0.0344	0.1165 ± 0.0208	0.8139 ± 0.0010	0.2621 ± 0.0036
Feature ablation	Without lncRNA structure	3	0.6586 ± 0.0294	0.1120 ± 0.0194	0.8169 ± 0.0007	0.2822 ± 0.0072
Feature ablation	Without lncRNA expression	3	0.6041 ± 0.0390	0.1156 ± 0.0185	0.7606 ± 0.0029	0.2315 ± 0.0029
Feature ablation	Disease ontology only	3	0.6572 ± 0.0304	0.1182 ± 0.0172	0.8123 ± 0.0020	0.2784 ± 0.0009
Feature ablation	lncRNA structure only	3	0.5609 ± 0.0230	0.0572 ± 0.0024	0.6120 ± 0.0016	0.0391 ± 0.0001
Feature ablation	lncRNA expression only	3	0.6414 ± 0.0290	0.1181 ± 0.0099	0.8125 ± 0.0015	0.2502 ± 0.0007
Node-embedding control	CDELDA-DT	5	0.6601 ± 0.0211	0.1173 ± 0.0130	0.8146 ± 0.0028	0.2843 ± 0.0024

Analysis	Model or feature configuration	Seeds	Both-cold AUROC	Both-cold AP	Disease-cold AUROC	Disease-cold AP
Node-embedding control	Learnable lncRNA embeddings	5	0.4823 ± 0.0204	0.0536 ± 0.0081	0.8034 ± 0.0033	0.2547 ± 0.0054
Node-embedding control	Learnable disease embeddings	5	0.4967 ± 0.0272	0.0597 ± 0.0026	0.4955 ± 0.0303	0.0638 ± 0.0097
Node-embedding control	Matrix factorization	5	0.4994 ± 0.0175	0.0513 ± 0.0051	0.5058 ± 0.0151	0.0322 ± 0.0010

Selected blocks are zeroed while retaining the 702- and 996-dimensional inputs and the same projection widths. Unmentioned blocks remain. Disease ontology includes the semantic and graph blocks. Disease text only here is a width-matched ablation, not the 768-input CDELDA-DT. AUROC and AP are disease-macro metrics.

The reference is CDELDA-DT. A learnable lncRNA or disease embedding replaces the corresponding content projection. MF replaces both. All use the same balanced objective and 500 updates. Held-out embeddings receive no association-based training signal. These controls are not matched for total parameter count. Three-seed ablations and five-seed controls are not interchangeable replicates.

Table S6. Sensitivity to k-core filtering.

Method	k	Both-cold AUROC	Both-cold AP	Disease-cold AUROC	Disease-cold AP
CDELDA	1	0.6974 ± 0.0415	0.1128 ± 0.0341	0.8339 ± 0.0012	0.2553 ± 0.0020
CDELDA	2	0.6594 ± 0.0424	0.1266 ± 0.0211	0.8171 ± 0.0010	0.2873 ± 0.0038
CDELDA	3	0.6592 ± 0.0137	0.1548 ± 0.0091	0.8032 ± 0.0034	0.3082 ± 0.0008
CDELDA	5	0.6188 ± 0.0253	0.1842 ± 0.0352	0.7863 ± 0.0016	0.3662 ± 0.0018
CDELDA-DT	1	0.7152 ± 0.0285	0.1127 ± 0.0086	0.8354 ± 0.0021	0.2507 ± 0.0034
CDELDA-DT	2	0.6558 ± 0.0272	0.1179 ± 0.0178	0.8159 ± 0.0017	0.2843 ± 0.0031
CDELDA-DT	3	0.6581 ± 0.0078	0.1645 ± 0.0151	0.8001 ± 0.0044	0.3096 ± 0.0029
CDELDA-DT	5	0.6128 ± 0.0250	0.1878 ± 0.0113	0.7851 ± 0.0020	0.3678 ± 0.0015
MF	1	0.4823 ± 0.0089	0.0343 ± 0.0016	0.4995 ± 0.0162	0.0181 ± 0.0009
MF	2	0.4975 ± 0.0170	0.0511 ± 0.0044	0.5082 ± 0.0074	0.0317 ± 0.0005
MF	3	0.4958 ± 0.0068	0.0703 ± 0.0026	0.4961 ± 0.0100	0.0481 ± 0.0025
MF	5	0.4970 ± 0.0073	0.1214 ± 0.0038	0.4967 ± 0.0147	0.0880 ± 0.0012
Popularity	1	0.5000 ± 0.0000	0.0214 ± 0.0005	0.5000 ± 0.0000	0.0143 ± 0.0000
Popularity	2	0.5000 ± 0.0000	0.0352 ± 0.0013	0.5000 ± 0.0000	0.0273 ± 0.0000
Popularity	3	0.5000 ± 0.0000	0.0479 ± 0.0014	0.5000 ± 0.0000	0.0407 ± 0.0000
Popularity	5	0.5000 ± 0.0000	0.0887 ± 0.0014	0.5000 ± 0.0000	0.0773 ± 0.0000

Disease-macro AUROC/AP use the same three matched seeds, full test candidates and fixed hyperparameters without retuning. Thus the 2-core values differ from five-seed main results. Changing k changes node composition, connectivity, positive prevalence and semantic input width together. Dataset sizes are given in main Table 1. This is a fixed-configuration sensitivity analysis, not a hyperparameter-retuning experiment.

Table S7. Sensitivity to unobserved-control definitions.

Analysis	Protocol	Model	Condition	Metric	Mean ± SD	Seeds
Control exclusion	Both-cold	CDELDA	Original	Disease-macro AUROC	0.6594 ± 0.0424	3

Analysis	Protocol	Model	Condition	Metric	Mean ± SD	Seeds
Control exclusion	Both-cold	CDELDA	Original	Disease-macro AP	0.1266 ± 0.0211	3
Control exclusion	Disease-cold	CDELDA	Original	Disease-macro AUROC	0.8171 ± 0.0010	3
Control exclusion	Disease-cold	CDELDA	Original	Disease-macro AP	0.2873 ± 0.0038	3
Control exclusion	Both-cold	CDELDA	Top 1% excluded	Disease-macro AUROC	0.6620 ± 0.0416	3
Control exclusion	Both-cold	CDELDA	Top 1% excluded	Disease-macro AP	0.1176 ± 0.0149	3
Control exclusion	Disease-cold	CDELDA	Top 1% excluded	Disease-macro AUROC	0.8170 ± 0.0012	3
Control exclusion	Disease-cold	CDELDA	Top 1% excluded	Disease-macro AP	0.2831 ± 0.0024	3
Control exclusion	Both-cold	CDELDA-DT	Original	Disease-macro AUROC	0.6558 ± 0.0272	3
Control exclusion	Both-cold	CDELDA-DT	Original	Disease-macro AP	0.1179 ± 0.0178	3
Control exclusion	Disease-cold	CDELDA-DT	Original	Disease-macro AUROC	0.8159 ± 0.0017	3
Control exclusion	Disease-cold	CDELDA-DT	Original	Disease-macro AP	0.2843 ± 0.0031	3
Control exclusion	Both-cold	CDELDA-DT	Top 1% excluded	Disease-macro AUROC	0.6543 ± 0.0255	3
Control exclusion	Both-cold	CDELDA-DT	Top 1% excluded	Disease-macro AP	0.1247 ± 0.0204	3
Control exclusion	Disease-cold	CDELDA-DT	Top 1% excluded	Disease-macro AUROC	0.8145 ± 0.0030	3
Control exclusion	Disease-cold	CDELDA-DT	Top 1% excluded	Disease-macro AP	0.2785 ± 0.0049	3
Control sampling	warm	CDELDA	1 per positive	Pooled AP	0.9345 ± 0.0011	5
Control sampling	warm	CDELDA	5 per positive	Pooled AP	0.7792 ± 0.0026	5
Control sampling	warm	CDELDA	10 per positive	Pooled AP	0.6675 ± 0.0037	5
Control sampling	warm	CDELDA	Full controls	Pooled AP	0.4182 ± 0.0054	5
Control sampling	warm	CDELDA-DT	1 per positive	Pooled AP	0.9339 ± 0.0011	5
Control sampling	warm	CDELDA-DT	5 per positive	Pooled AP	0.7773 ± 0.0021	5
Control sampling	warm	CDELDA-DT	10 per positive	Pooled AP	0.6655 ± 0.0028	5

Analysis	Protocol	Model	Condition	Metric	Mean ± SD	Seeds
Control sampling	warm	CDELDA-DT	Full controls	Pooled AP	0.4177 ± 0.0034	5
Control sampling	lncRNA-cold	CDELDA	1 per positive	Pooled AP	0.8978 ± 0.0017	5
Control sampling	lncRNA-cold	CDELDA	5 per positive	Pooled AP	0.6834 ± 0.0048	5
Control sampling	lncRNA-cold	CDELDA	10 per positive	Pooled AP	0.5446 ± 0.0071	5
Control sampling	lncRNA-cold	CDELDA	Full controls	Pooled AP	0.2834 ± 0.0082	5
Control sampling	lncRNA-cold	CDELDA-DT	1 per positive	Pooled AP	0.8983 ± 0.0018	5
Control sampling	lncRNA-cold	CDELDA-DT	5 per positive	Pooled AP	0.6842 ± 0.0030	5
Control sampling	lncRNA-cold	CDELDA-DT	10 per positive	Pooled AP	0.5454 ± 0.0043	5
Control sampling	lncRNA-cold	CDELDA-DT	Full controls	Pooled AP	0.2842 ± 0.0053	5
Control sampling	disease-cold	CDELDA	1 per positive	Pooled AP	0.7508 ± 0.0116	5
Control sampling	disease-cold	CDELDA	5 per positive	Pooled AP	0.4392 ± 0.0206	5
Control sampling	disease-cold	CDELDA	10 per positive	Pooled AP	0.3118 ± 0.0213	5
Control sampling	disease-cold	CDELDA	Full controls	Pooled AP	0.1390 ± 0.0142	5
Control sampling	disease-cold	CDELDA-DT	1 per positive	Pooled AP	0.7502 ± 0.0122	5
Control sampling	disease-cold	CDELDA-DT	5 per positive	Pooled AP	0.4487 ± 0.0168	5
Control sampling	disease-cold	CDELDA-DT	10 per positive	Pooled AP	0.3249 ± 0.0158	5
Control sampling	disease-cold	CDELDA-DT	Full controls	Pooled AP	0.1516 ± 0.0099	5
Control sampling	both-cold	CDELDA	1 per positive	Pooled AP	0.6575 ± 0.0145	5
Control sampling	both-cold	CDELDA	5 per positive	Pooled AP	0.2994 ± 0.0134	5
Control sampling	both-cold	CDELDA	10 per positive	Pooled AP	0.1833 ± 0.0094	5
Control sampling	both-cold	CDELDA	Full controls	Pooled AP	0.0637 ± 0.0042	5
Control sampling	both-cold	CDELDA-DT	1 per positive	Pooled AP	0.6600 ± 0.0214	5

Analysis	Protocol	Model	Condition	Metric	Mean ± SD	Seeds
Control sampling	both-cold	CDELDA-DT	5 per positive	Pooled AP	0.3080 ± 0.0226	5
Control sampling	both-cold	CDELDA-DT	10 per positive	Pooled AP	0.1918 ± 0.0177	5
Control sampling	both-cold	CDELDA-DT	Full controls	Pooled AP	0.0677 ± 0.0086	5
Label hiding	warm	CDELDA	0% hidden	Pooled AP	0.4182 ± 0.0054	5
Label hiding	warm	CDELDA	5% hidden	Pooled AP	0.3978 ± 0.0052	5
Label hiding	warm	CDELDA	10% hidden	Pooled AP	0.3771 ± 0.0054	5
Label hiding	warm	CDELDA	20% hidden	Pooled AP	0.3355 ± 0.0050	5
Label hiding	warm	CDELDA-DT	0% hidden	Pooled AP	0.4177 ± 0.0034	5
Label hiding	warm	CDELDA-DT	5% hidden	Pooled AP	0.3972 ± 0.0031	5
Label hiding	warm	CDELDA-DT	10% hidden	Pooled AP	0.3766 ± 0.0035	5
Label hiding	warm	CDELDA-DT	20% hidden	Pooled AP	0.3351 ± 0.0031	5
Label hiding	lncRNA-cold	CDELDA	0% hidden	Pooled AP	0.2834 ± 0.0082	5
Label hiding	lncRNA-cold	CDELDA	5% hidden	Pooled AP	0.2696 ± 0.0079	5
Label hiding	lncRNA-cold	CDELDA	10% hidden	Pooled AP	0.2559 ± 0.0076	5
Label hiding	lncRNA-cold	CDELDA	20% hidden	Pooled AP	0.2274 ± 0.0070	5
Label hiding	lncRNA-cold	CDELDA-DT	0% hidden	Pooled AP	0.2842 ± 0.0053	5
Label hiding	lncRNA-cold	CDELDA-DT	5% hidden	Pooled AP	0.2703 ± 0.0051	5
Label hiding	lncRNA-cold	CDELDA-DT	10% hidden	Pooled AP	0.2565 ± 0.0049	5
Label hiding	lncRNA-cold	CDELDA-DT	20% hidden	Pooled AP	0.2280 ± 0.0045	5
Label hiding	disease-cold	CDELDA	0% hidden	Pooled AP	0.1390 ± 0.0142	5
Label hiding	disease-cold	CDELDA	5% hidden	Pooled AP	0.1324 ± 0.0135	5
Label hiding	disease-cold	CDELDA	10% hidden	Pooled AP	0.1255 ± 0.0128	5
Label hiding	disease-cold	CDELDA	20% hidden	Pooled AP	0.1117 ± 0.0115	5
Label hiding	disease-cold	CDELDA-DT	0% hidden	Pooled AP	0.1516 ± 0.0099	5
Label hiding	disease-cold	CDELDA-DT	5% hidden	Pooled AP	0.1443 ± 0.0093	5
Label hiding	disease-cold	CDELDA-DT	10% hidden	Pooled AP	0.1367 ± 0.0088	5
Label hiding	disease-cold	CDELDA-DT	20% hidden	Pooled AP	0.1217 ± 0.0080	5
Label hiding	both-cold	CDELDA	0% hidden	Pooled AP	0.0637 ± 0.0042	5
Label hiding	both-cold	CDELDA	5% hidden	Pooled AP	0.0606 ± 0.0039	5
Label hiding	both-cold	CDELDA	10% hidden	Pooled AP	0.0576 ± 0.0040	5
Label hiding	both-cold	CDELDA	20% hidden	Pooled AP	0.0516 ± 0.0041	5
Label hiding	both-cold	CDELDA-DT	0% hidden	Pooled AP	0.0677 ± 0.0086	5
Label hiding	both-cold	CDELDA-DT	5% hidden	Pooled AP	0.0645 ± 0.0083	5
Label hiding	both-cold	CDELDA-DT	10% hidden	Pooled AP	0.0613 ± 0.0082	5
Label hiding	both-cold	CDELDA-DT	20% hidden	Pooled AP	0.0550 ± 0.0079	5

An auxiliary CDELDA-DT model supplies out-of-fold scores through three-way pair cross-fitting within each outer training mask. Exclude floor(0.01 × unobserved training pairs), with identifier-based tie breaking, before refitting. Excluded pairs are not relabeled positive. Test labels/masks remain unchanged. The selected pairs are not verified false negatives. AUROC and AP for control exclusion are disease-macro metrics.

For control sampling, conditions specify the number of unobserved controls per positive. Ten draws are averaged within each fold, using shared sampled pairs. All requested ratios are attainable in the main 2-core benchmark. Full controls means all unobserved pairs allowed by the fixed mask and exclusion policy. Label hiding uses ten nested draws to relabel the specified fraction of observed positives as unobserved while predictions and candidate masks remain fixed. Hidden fractions are not estimates of biological false-negative prevalence. Pooled AP avoids changing the disease-query denominator.

Table S8. Disease-input perturbations and ranking similarity.

Analysis	Protocol	Model	Condition	Metric	Mean ± SD	Seeds
Text perturbation	Both-cold	CDELDA	Original	Disease-macro AUROC	0.6594 ± 0.0424	3
Text perturbation	Both-cold	CDELDA	Original	Disease-macro AP	0.1266 ± 0.0211	3
Text perturbation	Disease-cold	CDELDA	Original	Disease-macro AUROC	0.8171 ± 0.0010	3
Text perturbation	Disease-cold	CDELDA	Original	Disease-macro AP	0.2873 ± 0.0038	3
Text perturbation	Both-cold	CDELDA	Zeroed text	Disease-macro AUROC	0.6572 ± 0.0304	3
Text perturbation	Both-cold	CDELDA	Zeroed text	Disease-macro AP	0.1182 ± 0.0172	3
Text perturbation	Disease-cold	CDELDA	Zeroed text	Disease-macro AUROC	0.8123 ± 0.0020	3
Text perturbation	Disease-cold	CDELDA	Zeroed text	Disease-macro AP	0.2784 ± 0.0009	3
Text perturbation	Both-cold	CDELDA	Shuffled text	Disease-macro AUROC	0.6583 ± 0.0351	3
Text perturbation	Both-cold	CDELDA	Shuffled text	Disease-macro AP	0.1226 ± 0.0225	3
Text perturbation	Disease-cold	CDELDA	Shuffled text	Disease-macro AUROC	0.8168 ± 0.0031	3
Text perturbation	Disease-cold	CDELDA	Shuffled text	Disease-macro AP	0.2869 ± 0.0037	3
Text perturbation	Both-cold	CDELDA-DT	Original	Disease-macro AUROC	0.6558 ± 0.0272	3
Text perturbation	Both-cold	CDELDA-DT	Original	Disease-macro AP	0.1179 ± 0.0178	3
Text perturbation	Disease-cold	CDELDA-DT	Original	Disease-macro AUROC	0.8159 ± 0.0017	3
Text perturbation	Disease-cold	CDELDA-DT	Original	Disease-macro AP	0.2843 ± 0.0031	3
Text perturbation	Both-cold	CDELDA-DT	Zeroed text	Disease-macro AUROC	0.6660 ± 0.0222	3

Analysis	Protocol	Model	Condition	Metric	Mean ± SD	Seeds
Text perturbation	Both-cold	CDELDA-DT	Zeroed text	Disease-macro AP	0.1419 ± 0.0281	3
Text perturbation	Disease-cold	CDELDA-DT	Zeroed text	Disease-macro AUROC	0.8183 ± 0.0009	3
Text perturbation	Disease-cold	CDELDA-DT	Zeroed text	Disease-macro AP	0.3013 ± 0.0014	3
Text perturbation	Both-cold	CDELDA-DT	Shuffled text	Disease-macro AUROC	0.6555 ± 0.0180	3
Text perturbation	Both-cold	CDELDA-DT	Shuffled text	Disease-macro AP	0.1262 ± 0.0219	3
Text perturbation	Disease-cold	CDELDA-DT	Shuffled text	Disease-macro AUROC	0.8112 ± 0.0013	3
Text perturbation	Disease-cold	CDELDA-DT	Shuffled text	Disease-macro AP	0.2819 ± 0.0033	3
Input reassignment	both-cold	CDELDA	Original input	Disease-macro AUROC	0.6617 ± 0.0312	5
Input reassignment	both-cold	CDELDA	Reassigned input	Disease-macro AUROC	0.6489 ± 0.0282	5
Input reassignment	both-cold	CDELDA	Original − reassigned	Disease-macro AUROC	0.0129 ± 0.0039	5
Input reassignment	both-cold	CDELDA	Original input	Disease-macro AP	0.1285 ± 0.0172	5
Input reassignment	both-cold	CDELDA	Reassigned input	Disease-macro AP	0.1243 ± 0.0159	5
Input reassignment	both-cold	CDELDA	Original − reassigned	Disease-macro AP	0.0043 ± 0.0051	5
Input reassignment	both-cold	CDELDA-DT	Original input	Disease-macro AUROC	0.6601 ± 0.0211	5
Input reassignment	both-cold	CDELDA-DT	Reassigned input	Disease-macro AUROC	0.6568 ± 0.0207	5
Input reassignment	both-cold	CDELDA-DT	Original − reassigned	Disease-macro AUROC	0.0033 ± 0.0018	5
Input reassignment	both-cold	CDELDA-DT	Original input	Disease-macro AP	0.1173 ± 0.0130	5
Input reassignment	both-cold	CDELDA-DT	Reassigned input	Disease-macro AP	0.1161 ± 0.0134	5
Input reassignment	both-cold	CDELDA-DT	Original − reassigned	Disease-macro AP	0.0012 ± 0.0021	5
Input reassignment	disease-cold	CDELDA	Original input	Disease-macro AUROC	0.8168 ± 0.0008	5
Input reassignment	disease-cold	CDELDA	Reassigned input	Disease-macro AUROC	0.8105 ± 0.0005	5
Input reassignment	disease-cold	CDELDA	Original − reassigned	Disease-macro AUROC	0.0063 ± 0.0010	5

Analysis	Protocol	Model	Condition	Metric	Mean ± SD	Seeds
Input reassignment	disease-cold	CDELDA	Original input	Disease-macro AP	0.2871 ± 0.0030	5
Input reassignment	disease-cold	CDELDA	Reassigned input	Disease-macro AP	0.2791 ± 0.0026	5
Input reassignment	disease-cold	CDELDA	Original − reassigned	Disease-macro AP	0.0080 ± 0.0019	5
Input reassignment	disease-cold	CDELDA-DT	Original input	Disease-macro AUROC	0.8146 ± 0.0028	5
Input reassignment	disease-cold	CDELDA-DT	Reassigned input	Disease-macro AUROC	0.8124 ± 0.0027	5
Input reassignment	disease-cold	CDELDA-DT	Original − reassigned	Disease-macro AUROC	0.0022 ± 0.0002	5
Input reassignment	disease-cold	CDELDA-DT	Original input	Disease-macro AP	0.2843 ± 0.0024	5
Input reassignment	disease-cold	CDELDA-DT	Reassigned input	Disease-macro AP	0.2812 ± 0.0029	5
Input reassignment	disease-cold	CDELDA-DT	Original − reassigned	Disease-macro AP	0.0031 ± 0.0009	5
Ranking similarity	both-cold	CDELDA	Common candidates	Top-20 Jaccard	0.6993 ± 0.0223	5
Ranking similarity	both-cold	CDELDA	Common candidates	Top-20 overlap	0.8048 ± 0.0186	5
Ranking similarity	both-cold	CDELDA	Common candidates	Score Spearman ϱ	0.9083 ± 0.0112	5
Ranking similarity	both-cold	CDELDA-DT	Common candidates	Top-20 Jaccard	0.8071 ± 0.0312	5
Ranking similarity	both-cold	CDELDA-DT	Common candidates	Top-20 overlap	0.8848 ± 0.0212	5
Ranking similarity	both-cold	CDELDA-DT	Common candidates	Score Spearman ϱ	0.9598 ± 0.0089	5
Ranking similarity	disease-cold	CDELDA	Common candidates	Top-20 Jaccard	0.7597 ± 0.0242	5
Ranking similarity	disease-cold	CDELDA	Common candidates	Top-20 overlap	0.8514 ± 0.0170	5
Ranking similarity	disease-cold	CDELDA	Common candidates	Score Spearman ϱ	0.9124 ± 0.0137	5
Ranking similarity	disease-cold	CDELDA-DT	Common candidates	Top-20 Jaccard	0.8621 ± 0.0192	5
Ranking similarity	disease-cold	CDELDA-DT	Common candidates	Top-20 overlap	0.9217 ± 0.0118	5
Ranking similarity	disease-cold	CDELDA-DT	Common candidates	Score Spearman ϱ	0.9641 ± 0.0065	5

Reassign text separately within training and held-out disease sets, or set it to zero, then retrain. Original values use the same three-seed subset. AUROC and AP are disease-macro metrics.

Ten permutations reassign complete held-out input vectors with no self-assignment. Each permutation uses a fresh forward pass, without refitting. Labels/masks are fixed. Paired original-minus-reassigned differences are calculated before rounding.

Disease pairs use common test candidates. Top-20 overlap is the shared-candidate fraction, whereas Jaccard is intersection/union. Pair summaries are averaged within folds and then across folds and seeds. These descriptive results assess ranking similarity, not independent biological specificity.

Table S9. Exploratory paired comparisons in both-cold evaluation.

Comparator	ΔAUROC	Approximate familywise AUROC interval	Adjusted p	ΔAP	Unadjusted 95% AP interval
CDELDA-DT	0.0017	[-0.0094, 0.0127]	1.0000	0.0112	[0.0024, 0.0222]
DSCMF*	0.1158	[0.0678, 0.1620]	0.0006	0.0749	[0.0382, 0.0996]
IPCARF*	0.1617	[0.1295, 0.1938]	0.0006	0.0934	[0.0517, 0.1132]
SIMCLDA*	0.0494	[0.0104, 0.0876]	0.0078	-0.0063	[-0.0400, 0.0227]
VGAELDA†	0.1617	[0.1295, 0.1938]	0.0006	0.0934	[0.0517, 0.1132]
LDA-VGHB*	0.1443	[0.1078, 0.1802]	0.0006	0.0755	[0.0391, 0.0999]

Δ is CDELDA minus comparator. AUROC intervals and centered-bootstrap p-values use Bonferroni adjustment across six comparisons. AP intervals are unadjusted. These approximate conditional intervals use 9,999 shared product-weight replicates over lncRNA groups and diseases with fixed predictions. They exclude retraining and benchmark-construction variability. Numerical checks do not establish interval coverage. Main Tables 2–3 report model means and seed-level SD.

Table S10. Candidate-level literature assessments.

Disease	Rank / lncRNA	Evidence / access	Scope of reviewed evidence	Source identifier
Breast cancer	1. MIR210HG	Functional<br>Full text	Breast-cell lncRNA perturbation and tissue findings support a functional association, not breast specificity.	PMID: 35346372<br>PMID: 31399552
Breast cancer	2. ESRG	Observational<br>Full text	Public-cohort BRCA expression and survival associations, without ESRG perturbation.	PMID: 39310490
Breast cancer	3. MEG8	Functional<br>Full text	Human breast-cell MEG8 overexpression with growth and tumor assays, distinguished from mouse Rian.	PMID: 39706842
Breast cancer	4. LINC02562	Observational<br>Full text	Expression differs between inflammatory and non-inflammatory breast tumors, not cancer versus normal.	PMID: 42066072
Breast cancer	5. NUTM2A-AS1	Unconfirmed<br>Full text	The retrieved functional report was retracted and excluded. No alternative direct support was identified.	—
Breast cancer	6. DNM3OS	Unconfirmed<br>Abstract	Mouse Dnm3os and human miR-214 findings could not be attributed directly to human DNM3OS.	PMID: 27693451
Breast cancer	7. DLEU2	Functional<br>Full text	Patient samples and breast-cell DLEU2 knockdown, proliferation, stemness and EMT assays.	PMID: 38296962
Breast cancer	8. FAM30A	Observational<br>Full text	KIAA0125 is FAM30A. Public-cohort expression and outcome associations, without gene perturbation.	PMID: 41670092

Disease	Rank / lncRNA	Evidence / access	Scope of reviewed evidence	Source identifier
Breast cancer	9. SLCO4A1-AS1	Observational Full text	BRCA expression is reported within a lung-cancer study, without a breast-specific functional experiment.	PMID: 37726723
Breast cancer	10. GATA6-AS1	Observational Full text	Expression after SIRT7 knockdown or drug treatment, not GATA6-AS1 perturbation. Partial SinoMed material was not accepted.	PMID: 29123410 PMID: 29795261
Prostate cancer	1. MEG8	Observational Full text	Individual prostate-cancer versus normal SRA expression (FDR threshold 0.10). BPH evidence is only indirect.	PMID: 31694982 PMID: 27634882
Prostate cancer	2. MIR210HG	Functional Abstract	Cell/animal functional findings were checked in the complete abstract, not independently in full text.	PMID: 37758909
Prostate cancer	3. ESRG	Unconfirmed Full text	The pan-cancer source lacks a significant PRAD expression difference. Cell-line expression alone was not accepted.	PMID: 39310490
Prostate cancer	4. LINC02562	Indirect Excerpt	Listed in preliminary screening in an author response. The article investigates MIR22HG, not individual LINC02562 function.	Peer-review file for PMID: 40530115†
Prostate cancer	5. DNM3OS	Indirect Full text	Functional findings concern benign prostatic hyperplasia and stromal transition, not prostate cancer.	PMID: 31694982
Prostate cancer	6. NUTM2A-AS1	Functional Abstract	Patient tissue and DU145 perturbation in the complete abstract, separate from the retracted breast-cancer report.	PMID: 38583010
Prostate cancer	7. GATA6-AS1	Observational Full text	Tumor-associated versus normal prostate stroma (adjusted P threshold 0.10). Inconsistent reporting precludes assigning direction.	DOI: 10.3390/informatics11020014
Prostate cancer	8. SNHG18	Functional Abstract	Abstract-level function involves human expression and mouse cell/bone-metastasis models. Separate full-text evidence is observational.	PMID: 41702447 PMID: 37644825
Prostate cancer	9. LUADT1	Unconfirmed Search only	No admissible individual LUADT1–prostate-cancer support identified. This is not evidence of biological absence.	—
Prostate cancer	10. MIR100HG	Observational Full text	Individual prostate expression association. Panel performance is not individual validation. MIR100HG was not among four qPCR targets.	PMID: 36911392

Categories match main Table 4. Functional requires lncRNA manipulation. Observational includes individual expression or clinical association. Indirect does not directly support the candidate association. Unconfirmed denotes missing direct support or unresolved attribution, not a biological negative. Access describes the material supporting the displayed category. Retracted reports are excluded from support.

† The linked publisher peer-review file for PMID 40530115 lists LINC02562 in the preliminary screening reply. The published study concerns MIR22HG. PMID 41702447 supplies abstract-level functional evidence for SNHG18, whereas PMID 37644825 supplies full-text observational evidence.

Case-study predictions use disease-cold seed 41001, fold 0. The two fixed top-ten lists contain 13 unique lncRNAs, seven shared. Scores for 1,227 common unobserved candidates have Spearman $\rho = 0.955$. Top-20 lists share 16 candidates (Jaccard 0.667). Candidate selection was not revised after literature review. Literature support is not independent experimental validation.

Source identifiers link to reviewed publications or related materials. Listing a source does not necessarily indicate support for the candidate association. Evidence scope and source access are specified for each candidate.
